# Counseling received in prenatal care by Brazilian women

**DOI:** 10.1590/1980-220X-REEUSP-2024-0198en

**Published:** 2025-04-25

**Authors:** Ana Clara Alves Tomé de Souza, Marina Garcia Avelar Rocha, Kênia Lara Silva, Rafaela Siqueira Costa Schreck, Eunice Francisca Martins, Nágela Cristine Pinheiro Santos, Mariana Santos Felisbino-Mendes

**Affiliations:** 1 Universidade Federal de Minas Gerais, Escola de Enfermagem, Belo Horizonte, MG, Brazil.; 2 Universidade Federal de Minas Gerais, Escola de Enfermagem, Departamento de Enfermagem Aplicada, Belo Horizonte, MG, Brazil.; 3 Universidade Federal de Minas Gerais, Escola de Enfermagem, Departamento de Enfermagem Materno-Infantil e Saúde Pública, Belo Horizonte, MG, Brazil

**Keywords:** Health Education, Prenatal Care, Socioeconomic Factors, Women’s Health, Health Inequities

## Abstract

**Objective:**

To analyze the counseling received during prenatal care by Brazilian women and its association with sociodemographic and healthcare characteristics.

**Method:**

A cross-sectional study using data from the Brazilian National Health Survey2013 and 2019 editions. Women aged 18 to 49 who had prenatal care up to two years prior to the survey (1,851 in 2013 and 2,819 in 2019) were studied. Prevalence rates were estimated with their 95% Confidence Intervals for the different guidelines. Prevalence rates, unadjusted Prevalence Ratio and 95% Confidence Interval were then estimated according to sociodemographic and healthcare variables.

**Results:**

The results reveal inequalities in the counseling received during prenatal care, especially among women living in less developed regions of the country, with brown/black skin color, low levels of education, and who receive prenatal care in the public health system.

**Conclusion:**

Counseling is provided by professionals, with emphasis on counseling on referral maternity, but there are inequities in the provision of this counseling.

## INTRODUCTION

Obstetric care should be based on interprofessional care provided to women during prenatal care, childbirth and the postpartum period. Women need to be included as leading actors of this care and their choices, with access to screening, diagnosis and prevention of diseases, in addition to information on physiological and behavioral aspects, based on scientific evidence, in addition to support in relation to social and emotional issues^(1)^. Thus, the use of soft technologies, such as qualified listening and humanization of care^(2)^, which are inherent to good obstetric practices, can contribute to women experiencing motherhood positively, which involves self-esteem, competency and autonomy, arising from safety, respect and dignity, through communication, support and health education^(1)^.

In this regard, health education is considered a social practice and the guiding principle of prenatal care^(3)^. From this perspective, it is understood that all appointments are opportunities to carry out individual or collective educational interventions, which have also been recommended to repair vulnerabilities during pregnancy^(4,5)^. To this end, health education assumes a humanistic and transformative commitment that, under a Freirean conception, must be supported by dialogue between educator and student, in a multidimensional process that considers cultural, economic and social aspects^(6)^. However, studies have identified that healthcare professionals have not carried out or have carried out, partially, health education in the care of pregnant women^(7,8)^. When assessing the quality of prenatal care in Brazil’s basic health network, it was found that only 60% of pregnant women received counseling regarding prenatal care, such as exclusive breastfeeding, nutrition and weight gain, newborn care and the importance of Pap smear^(9)^. Other findings also identified limited access to counseling on childbirth^(10)^ and on the reference maternity hospital^(11)^, indicating a deficit in relation to health education provided during prenatal care. When the completeness of recommended actions and counseling during prenatal care was identified, these were significantly higher for older pregnant women with higher income, and in larger municipalities^(9)^. This finding reveals that social and individual inequalities persist in the quality of prenatal care offered to women in the country, compromising the guarantee of the principle of equity within the Brazilian Health System (In Portuguese, *Sistema* Único *de Saúde* - SUS). Furthermore, care models focused on disease and technical procedures persist instead of a care model based on comprehensive health with health education actions^(12)^.

Thus, the counseling provided to pregnant women is an indicator that can assess the quality of prenatal care, given its direct association with health promotion. However, most studies found in literature assess the quality of prenatal care locally/regionally and only through access markers and/or clinical markers^(11,13-16)^. When assessed, the guidelines are considered in smaller numbers, often not identifying the subgroups most affected by the lack of this assistance, a fact that can also be observed in national studies^(12,15)^.

Considering the above, the study focused on the counseling provided to pregnant women during prenatal appointments. Studies of this nature can contribute to a better assessment of the reality of prenatal care at a population level, assessing other elements in addition to access markers. Thus, this study aimed to analyze the counseling received during prenatal care by Brazilian women and its association with sociodemographic and care characteristics.

## METHOD

### Study Design

This is a cross-sectional study with data from the Brazilian National Health Survey (In Portuguese, *Pesquisa Nacional de Saúde* - PNS), 2013 and 2019 editions, which is a Brazilian survey whose main objective is to characterize the health situation and lifestyles of the Brazilian population.

### Sample Definition

The PNS sampling process was carried out in three stages: in the first, census sectors were selected; in the second, households were selected; and in the third, for each household, a resident aged 18 or over in 2013 and 15 or over in 2019 was selected, all by simple random sampling. The PNS questionnaire, in both editions, was divided into modules that cover the characteristics of the household, all residents and the selected adult resident. In module S, prenatal care was investigated among women who gave birth from 07/28/2011 to 07/27/2013, in the 2013 edition, and in the period equal to or after 07/28/2017, in the 2019 edition, i.e., in the last two years before the surveys, considering only the last birth to answer the questions. Among the women who responded to module S of the questionnaires, a subsample was defined, including only women aged 18 to 49 who had prenatal care during their last pregnancy, totaling 1,851 women available for the study in 2013 and 2,819 women in 2019.

### Data Collection

In this study, ten specific questions were analyzed regarding the guidelines provided during prenatal care, as follows: during prenatal appointments, did you receive any of the following guidelines: not missing appointments, maintain a healthy diet, not to smoke, not to drink, about breastfeeding, signs of pregnancy risk, and signs of labor, risks of high blood pressure, risk of high blood sugar and which healthcare service should you go to at the time of delivery?

However, it was observed that, compared to 2013, only one of the guidelines previously analyzed was maintained in 2019, which corresponds to the counseling on which reference healthcare service the woman should go to at the time of delivery. The other guidelines were not investigated, hindering the monitoring of these indicators. The guidelines were considered as dependent variables, and the following sociodemographic variables were used as independent variables: age range (18–29 years, 30–39 years and 40–49 years); region (North, Northeast, Midwest, Southeast and South); education, in years (0–8 years, 9–11 years or 12 or more); skin color (white, yellow, brown, black and indigenous); and location of prenatal care (public health system, private or other location).

### Data Analysis and Collection

First, the prevalences with their 95% Confidence Intervals of the different guidelines received by women in 2013 and 2019 were estimated. Then, these prevalences were estimated according to sociodemographic and assistance variables. Pearson’s chi-square test was used to assess statistical differences between the prevalences. Finally, the unadjusted Prevalence Ratio (uPR) and the 95% Confidence Interval of having received counseling for the different groups of independent variables were estimated only for the factors that in descriptive analysis presented a p < 0.2. The data were analyzed using the statistical program Stata version 14.2 using the survey module to obtain population estimates, considering stratum, cluster and weight of individuals in the complex sample.

### Ethical Aspects

This study uses secondary data from the PNS publicly available on official websites of the Brazilian Ministry of Health or the Brazilian Institute of Geography and Statistics, and is exempt from analysis by a Research Ethics Committee, according to Resolution 510/2016 of the Brazilian National Health Council. The PNS was approved by the Brazilian National Research Ethics Committee in July 2013 and in August 2019.

## RESULTS

It is observed that in both 2013 and 2019, respectively, the majority of women who gave birth in the last two years before the surveys were aged between 18–29 years (61.2%/52.7%), lived in the Southeast region (38.1%/36.9%), followed by the Northeast (29.1%/29.5%), had education between 9–11 years (47.2%/8.6%), self-declared as brown (50.0%/51.7%) and used the public healthcare service for prenatal care (71.9%/70%) (Table 1).

**Table 1:** Prevalence and 95% Confidence Intervals of sociodemographic and healthcare characteristics of Brazilian women who received prenatal care in Brazilian National Health Survey – Brazil, 2013 and 2019.

Sociodemographic and assistance variables	2013		2019	
	n*	%** (95%CI)	n*	%** (95%CI)
**Age range (years)**				
18–29	1,120	61.2 (57.4–64.9)	1,456	52.7 (49.3–55.9)
30–39	662	35.2 (31.7–38.9)	1,124	41.5 (38.3–44.8)
40–49	69	3.6 (2.5–5.1)	149	5.7 (4.4–7.3)
**Region**				
Midwest	232	8.2 (7.0–9.6)	344	9.2 (7.9–10.8)
North	517	9.9 (8.5–11.5)	687	11.6 (10.3–13.1)
Northeast	565	29.1 (25.9–32.7)	1,041	29.5 (26.9–32.2)
Southeast	345	38.1 (34.1–42.3)	476	36.9 (33.4–40.5)
South	192	14.6 (12.1–17.5)	271	12.5 (10.8–14.5)
**Education (years)**				
0–8	707	36.7 (33.1–40.0)	900	27.6 (24.8–30.6)
9–11	830	47.2 (43.4–51.1)	1,310	48.6 (45.3–51.8)
12 or more	314	16.1 (13.5–19.1)	609	23.7 (21.0–26.6)
**Skin color/race**				
White	593	39.9 (36.1–43.8)	802	35.1 (32.0–38.3)
Black	167	8.8 (6.9–11.2)	293	11.8 (9.6–14.3)
Brown	1,052	50.0 (46.2–53.8)	1,665	51.7 (48.4–54.9)
Yellow	24	1.1 (0.006–2.1)	31	1.0 (0.6–1.5)
Indigenous	15	0.1 (0.0006–0.6)	28	0.6 (0.3–1.0)
**Location of prenatal care**				
**Public**	1,401	71.9 (68.0–75.5)	2,141	70.0 (66.7–73.1)
**Private**	435	27.3 (23.8–31.2)	677	29.9 (26.8–33.2)
**Others**	15	0.7 (0.3–1.5)	1	0.1 (0–1.0)

Note: *sample n; ** population prevalence; 95%CI: 95% Confidence Interval.

A significant increase in the prevalence of counseling on the reference maternity hospital was observed from 2013 (74.9%) to 2019 (83.0%) (Table 2). However, inequalities were observed in the provision of this counseling in both periods, since women aged 18–29, who lived in the North and Northeast regions, with low education, indigenous, black and brown women, who received prenatal care in the public health system, had a lower prevalence of being counseled.

**Table 2:** Prevalence, unadjusted Prevalence Ratios and 95% Confidence Intervals of general counseling received during prenatal care among Brazilian women who gave birth in the last two years before the surveys in Brazilian National Health Survey – Brazil, 2013 and 2019.

Sociodemographic and assistance variables	Reference maternity hospital/2013			Reference maternity hospital/2019			Not missing appointments		
	n*	%** (95%CI)	uPR (95%CI)	n*	%**(95%CI)	uPR (95%CI)	n*	%**(95%CI)	uPR (95%CI)
**Received counseling**	1,362	74.9 (71.4–78.1)	-	2,244	83.0 (80.7–85.1)	-	1,680	88.9 (85.8–91.4)	-
**Age range (years)**		p*** = 0.011			p*** = 0.0006			p*** = 0.338	
18–29	807	71.1 (66.3–75.5)	Ref.	1,113	79.0 (75.3–82.3)	Ref.	1,018	87.5 (83.1–90.9)	-
30–39	505	81.1 (76.0–85.4)	**1.14 (1.04–1.24)**	939	88.2 (84.9–90.8)	**1.08 (1.02–1.15)**	598	91.5 (87.4–94.3)	-
40–49	50	77.0 (58.3–88.9)	1.08 (0.87–1.33)	124	85.5 (75.0–92.1)	1.15 (0.99–1.34)	64	87.0 (63.7–96.3)	-
**Region**		p*** < 0.0001			p***< 0.0001			p***= 0.530	
Midwest	168	71.2 (62.9–78.2)	Ref.	298	87.9 (82.3–91.8)	Ref.	203	86.0 (79.5–90.7)	-
North	380	72.6 (64.9–79.1)	1.02 (0.88–1.18)	504	72.4 (66.6–77.6)	0.88 (0.80–0.98)	478	93.4 (89.1–96.1)	-
Northeast	368	61.0 (53.9–67.7)	0.85 (0.73–1.00)	781	75.9 (71.6–79.7)	0.87 (0.81–0.94)	516	90.0 (83.6–94.3)	-
Southeast	279	83.6 (77.5–88.3)	**1.17 (1.03–1.33)**	410	87.7 (82.6–91.5)	1.01 (0.93–1.08)	309	87.3 (81.0–91.7)	-
South	167	83.3 (74.2–89.7)	**1.17 (1.01–1.34)**	251	92.3 (87.6–95.4)	1.09 (1.00–1.18)	174	89.0 (79.8–94.3)	-
**Education (years)**		p*** = 0.051			p*** = 0.0002			p*** = 0.656	
0–8	491	69.4 (63.7–74.6)	Ref.	675	78.0 (73.6–81.9)	Ref.	652	90.4 (85.1–93.9)	-
9–11	620	77.7 (72.6–82.0)	**1.11 (1.01–1.23)**	1,026	81.7 (78.0–84.9)	1.02 (0.95–1.10)	754	87.7 (82.6–91.5)	-
12 or +	251	79.0 (69.6–86.0)	1.13 (1.00–1.29)	543	91.5 (87.0–94.5)	**1.11 (1.03–1.19)**	274	88.8 (82.0–93.2)	-
**Skin color/race**		p*** < 0.0001			p*** = 0.008			p*** = 0.735	
White	475	84.0 (79.2–87.8)	Ref.	683	88.7 (84.8–91.7)	Ref.	534	89.8 (85.0–93.2)	-
Black	116	72.6 (61.9–81.2)	1.09 (1.00–1.19)	238	84.3 (75.1–90.6)	1.04 (0.97–1.12)	156	87.6 (72.9–94.9)	-
Brown	743	68.5 (63.3–73.3)	**1.13 (1.07 –1.19)**	1,270	80.4 (77.2–83.3)	**1.07 (1.03–1.12)**	952	88.3 (83.9–91.6)	-
Yellow	17	79.2 (52.9–92.8)	1.04 (0.88–1.23)	25	86.0 (64.1–95.4)	1.02 (0.90–1.17)	24	100.0	-
Indigenous	11	54.3 (18.0–86.6)	1.26 (0.94–1.68)	25	98.0 (91.9–99.5)	**0.92 (0.88–0.96)**	14	97.3 (78.9–99.7)	-
**Location of prenatal care**	p*** < 0.0001			p*** = 0.002			p*** = 0.137	
Public	993	69.4 (65.0–73.5)	Ref.	1,619	79.9 (77.1–82.4)	Ref.	1,286	89.5 (85.9–92.3)	-
Private	356	88.7 (84.0–92.0)	**1.27 (1.18–1.38)**	614	90.3 (85.7–93.6)	**1.07 (1.01–1.13)**	384	88.0 (81.2–92.5)	-
Others	13	95.8 (80.7–99.2)	**1.38 (1.25–1.51)**	01	100.0	**1.18 (1.14–1.22)**	10	60.9 (23.5–88.8)	-

Note: *sample n; ** population prevalence; *** Pearson’s chi-square test; 95%CI: 95% Confidence Interval; uPR: unadjusted Prevalence Ratio.

Counseling on not missing appointments showed a high prevalence (88.9%). There was no association between the sociodemographic and assistance variables and this counseling. As for counseling related to lifestyle habits, such as maintaining a healthy diet, not smoking and not drinking, regardless of the sociodemographic and assistance variables, the prevalence of having received them was over 80%, and counseling on healthy eating was the most frequent among all of these (96.8%) (Table 3).

**Table 3:** Prevalence, unadjusted Prevalence Ratio and 95% Confidence Intervals of counseling received on lifestyle habits during prenatal care among Brazilian women who gave birth in the last two years before the surveys in Brazilian National Health Survey – Brazil, 2013 and 2019.

Sociodemographic and assistance variables	Healthy eating			Not smoking			Not drinking		
	n*	%** (95%CI)	uPR (95%CI)	n*	%**(95%CI)	uPR (95%CI)	n*	%**(95%CI)	uPR (95%CI)
**Received counseling**	1,792	96.8 (95.2–97.9)	-	1,684	89.1 (86.0–91.6)	-	1,687	89.9 (87.1–92.2)	-
**Age range (years)**		p*** = 0.491			p*** = 0.824			p*** = 0.291	
18–29	1,080	96.4 (94.0–97.8)	-	1,015	88.6 (84.7–91.6)	-	1,019	89.2 (85.4–92.2)	Ref.
30–39	643	97.2 (94.8–98.5)	-	603	89.7 (83.7–93.7)	-	602	90.3 (85.3–93.7)	1.02 (0.96–1.09)
40–49	69	100	-	66	92.8 (66.9–98.8)	-	66	97.5 (91.1–99.3)	1.00 (0.91–1.10)
**Region**		p*** = 0.361			p*** = 0.153			p*** = 0.559	
Midwest	226	96.4 (91.9–98.4)	-	211	88.6 (80.1–93.7)	Ref.	210	88.6 (80.2–93.7)	-
North	501	98.0 (96.3–99.0)	-	482	92.9 (86.5–96.3)	1.04 (0.96–1.14)	482	93.0 (86.9–96.4)	-
Northeast	548	98.0 (96.2–99.0)	-	516	92.4 (86.5–95.8)	1.04 (0.95–1.14)	513	91.5 (85.7–95.1)	-
Southeast	335	96.4 (92.6–98.3)	-	299	85.4 (79.0–90.1)	0.96 (0.87–1.06)	302	86.7 (82.1–91.7)	-
South	182	94.8 (87.6–97.9)	-	176	89.9 (81.5–94.8)	1.01 (0.91–1.12)	180	91.0 (81.8–95.8)	-
**Education (years)**		p*** = 0.072			p*** = 0.970			p*** = 0.706	
0–8	685	97.1 (94.8–98.4)	Ref.	646	88.8 (93.5–92.5)	-	648	88.7 (83.4–92.5)	-
9–11	798	95.8 (92.6–97.6)	0.98 (0.95–1.01)	754	89.5 (84.8–92.8)	-	755	91.0 (87.3–93.7)	-
12 or +	309	99.2 (97.5–99.8)	1.02 (1.00–1.04)	284	88.9 (79.8–94.2)	-	284	89.3 (80.5–94.4)	-
**Skin color/race**		p*** = 0.890			p*** = 0.494			p*** = 0.258	
White	574	96.3 (92.7–98.2)	-	535	90.1 (85.9–93.1)	-	541	91.9 (88.0–94.6)	-
Black	162	97.1 (92.1–99.0)	-	151	90.9 (79.7–96.2)	-	149	90.3 (79.4–95.8)	-
Brown	1,018	97.0 (94.8–98.2)	-	960	87.3 (82.0–91.1)	-	959	87.6 (83.0–91.1)	-
Yellow	24	100.0	-	24	100.0	-	24	100.0	-
Indigenous	14	97.3 (78.9–99.7)	-	14	97.3 (78.9–99.7)	-	14	97.3 (78.9–99.7)	-
**Location of prenatal care**	p*** = 0,105			p*** = 0.542			p*** = 0.116	
Public	1,352	96.2 (94.0–97.6)	Ref.	1,278	89.0 (85.3–91.8)	-	1,277	88.8 (85.1–91.7)	Ref.
Private	426	98.2 (95.8–99.3)	1.02 (0.99–1.04)	392	89.2 (82.4–93.6)	-	396	92.5 (88.0–95.3)	1.04 (0.98–1.09)
Others	18	99.4 (95.3–99.9)	1.03 (1.01–1.05)	14	99.4 (95.3–99.9)	-	14	99.4 (95.3–99.9)	1.11 (1.07–1.16)

Note: *sample n; ** population prevalence; *** Pearson’s chi-square test; 95%CI: 95% Confidence Interval; uPR: unadjusted Prevalence Ratio.

It is also observed that the prevalence of counseling on the risk of high blood pressure was 91.2% and the risk of high blood sugar was 92.9%, demonstrating that they are frequent for most women who had high blood pressure and altered blood sugar levels during pregnancy, since only those who had these alterations were asked about these guidelines (Table 4). There was no association between sociodemographic and assistance variables and receiving counseling on lifestyle habits and risks during the gestational period described above, with the exception of counseling on the risk of high blood sugar, in which prenatal care provided in the public health system is associated with a lower prevalence of receiving such counseling (Tables 3 and 4).

**Table 4:** Prevalence, unadjusted Prevalence Ratio and 95% Confidence Intervals of counseling received regarding risks during pregnancy due to high blood pressure and blood glucose levels among Brazilian women who gave birth in the last two years before the surveys in Brazilian National Health Survey – Brazil, 2013 and 2019.

Sociodemographic and assistance variables	Risk of high blood pressure			Risk of high blood sugar		
	n*	%** (95%CI)	uPR (95%CI)	n*	%**(95%CI)	uPR (95%CI)
**Received counseling**	347	91.2 (85.7–94.7)	-	89	92.9 (86.0–96.5)	-
**Age range (years)**		p*** = 0.719			p*** = 0.671	
18–29	191	90.0 (82.0–94.7)	-	42	90.6 (78.5–96.2)	-
30–39	134	92.6 (83.0–96.9)	-	40	94.1 (80.9–98.4)	-
40–49	22	92.7 (69.8–98.6)	-	7	100.0	-
**Region**		p*** = 0.610			p*** = 0.177	
Midwest	50	94.1 (82.1–98.3)	-	8	77.0 (38.0–94.8)	Ref.
North	102	89.9 (81.7–94.7)	-	27	87.7 (69.9–95.7)	1.14 (0.75–1.72)
Northeast	106	94.2 (88.7–97.1)	-	18	92.1 (68.2–98.5)	1.19 (0.79–1.80)
Southeast	51	90.6 (78.3–96.3)	-	19	95.7 (82.2–99.1)	1.24 (0.83–1.84)
South	38	85.7 (58.3–96.5)	-	17	98.9 (91.7–99.8)	1.28 (0.86–7.89)
**Education (years)**		p*** = 0.921			p*** = 0.553	
0–8	145	90.0 (79.7–95.4)	-	29	91.8 (76.7–97.4)	-
9–11	150	92.0 (83.6–96.3)	-	36	91.3 (79.0–96.7)	-
12 or +	52	91.1 (70.5–97.8)	-	24	100.0	-
**Skin color/race**		p*** = 0.473			p*** = 0.936	
White	103	87.6 (74.7–94–4)	-	27	92.5 (73.1–98.2)	-
Black	31	93.6 (80.8–98.1)	-	4	88.8 (45.0–98.7)	-
Brown	203	92.9 (85.5–96.6)	-	54	92.7 (83.7–96.9)	-
Yellow	4	100.0	-	3	100.0	-
Indigenous	6	70.7 (14.9–97.1)	-	1	100.0	-
**Location of prenatal care**		p*** = 0,092			p***<0.0001	
Public	275	89.4 (82.5–93.7)	Ref.	61	91.0 (81.4–95.9)	Ref.
Private	71	96.6 (82.9–99.4)	1.08 (0.99–1.17)	28	99.0 (92.6–99.9)	1.17 (1.08–1.28)
Others	01	41.1 (4.2–91.8)	0.46 (0.08–2.35)	0	0	-

Note: *sample n; ** population prevalence; *** Pearson’s chi-square test; 95%CI: 95% Confidence Interval; uPR: unadjusted Prevalence Ratio.

In relation to all the guidelines studied, a lower prevalence of specific guidelines on the pregnancy-puerperal cycle was observed, such as signs of labor (70.8%) and signs of risk in pregnancy (75.2%), with counseling on signs of labor being the least prevalent (Tables 2 and 5). Furthermore, it is observed that some population subgroups received less specific counseling on pregnancy than others, such as black, brown, yellow and indigenous women, with 0–8 years of education, who lived in the Northeast region and received prenatal care in the public health system (Table 5), as was observed in the reference maternity hospital. It is noteworthy that, when such counseling is related to education, the prevalence is 15%, lower among women with low schooling compared to those with high education (Table 5).

**Table 5:** Prevalence, unadjusted Prevalence Ratio and 95% Confidence Intervals of specific counseling on the pregnancy-puerperal cycle received during prenatal care by Brazilian women who gave birth up to two years before the survey in Brazilian National Health Survey – Brazil, 2013 and 2019.

Sociodemographic and assistance variables	Signs of labor			Signs of pregnancy risk			Breastfeeding		
	n*	%** (95%CI)	uPR (95%CI)	n*	%**(95%CI)	uPR (95%CI)	n*	%**(95%CI)	uPR (95%CI)
**Received counseling**	1,320	70.8 (67.3–74.2)	-	1,403	75.2 (71.7–78.4)	-	1,543	82.4 (79.7–85.2)	-
**Age range (years)**		p*** = 0.048			p*** = 0.019			p*** = 0.123	
18–29	796	68.7 (63.9–73.2)	Ref.	831	72.0 (67.1–76.3)	Ref.	932	80.0 (75.5–83.8)	Ref.
30–39	476	75.9 (70.4–80.6)	1.10 (1.00–1.22)	517	79.9 (74.6–84.4)	1.11 (1.01–1.21)	553	86.4 (81.6–90.2)	1.08 (1.00–1.16)
40–49	48	57.8 (38.8–74.7)	0.84 (0.60–1.17)	55	84.2 (70.8–92.2)	1.17 (1.01–1.34)	58	82.4 (79.0–85.2)	1.03 (0.85–1.25)
**Region**		p*** = 0.028			p*** = 0.088			p*** = 0.288	
Midwest	155	67.4 (59.9–74.3)	Ref.	175	74.1 (65.9–80.9)	Ref.	184	80.2 (73.0–85.9)	-
North	360	68.4 (60.6–75.4)	1.01 (0.87–1.18)	392	75.5 (67.8–81.9)	1.01 (0.88–1.17)	422	78.7 (69.6–85.6)	-
Northeast	393	64.2 (57.5–70.5)	0.95 (0.82–1.10)	411	68.8 (62.0–74.8)	0.92 (0.90–1.06)	469	78.8 (72.4–84.1)	-
Southeast	261	74.7 (67.9–80.4)	1.10 (0.96–1.26)	270	78.5 (71.9–83.9)	1.05 (0.93–1.20)	295	85.1 (78.9–89.7)	-
South	151	77.5 (69.1–84.2)	1.14 (0.99–1.32)	155	79.6 (70.3–86.6)	1.07 (0.92–1.24)	173	85.9 (76.5–92.0)	-
**Education (years)**		p*** < 0.0001			p*** = 0.029			p*** = 0.0002	
0–8	467	61.6 (55.7–67.1)	Ref.	514	69.9 (64.0–75.1)	Ref.	559	75.7 (69.8–80.7)	Ref.
9–11	616	74.2 (68.7–79.0)	**1.20 (1.07–1.35)**	635	77.0 (71.9–81.5)	1.10 (0.99–1.21)	710	84.2 (79.2–88.2)	**1.11 (1.01–1.21)**
12 or +	237	82.0 (75.7–87.0)	**1.33 (1.18–1.49)**	254	81.9 (73.6–78.4)	1.17 (1.04–1.31)	274	92.1 (87.5–95.1)	**1.21 (1.12–1.32)**
**Skin color/race**		p*** = 0.0001			p*** = 0.015			p*** = 0.012	
White	444	79.3 (74.2–83.6)	Ref.	467	80.9 (75.6–85.3)	Ref.	514	88.1 (83.3–91.7)	Ref.
Black	115	60.8 (48.4–71.9)	**1.15 (1.05–1.27)**	123	65.4 (52.6–76.3)	**1.13 (1.02–1.25)**	140	75.2 (61.7–85.1)	1.12 (1.00–1.23)
Brown	736	67.0 (61.9–71.6)	**1.10 (1.04–1.16)**	782	72.5 (67.4–77.1)	**1.07 (1.01–1.13)**	857	79.3 (74.8–83.2)	**1.08 (1.03–1.13)**
Yellow	17	42.5 (17.9–71.6)	**1.30 (1.07–1.58)**	19	58.8 (28.2–77.1)	1.19 (0.95–1.49)	20	77.2 (48.7–92.4)	1.10 (0.91–1.32)
Indigenous	8	34.8 (12.6–66.5)	**1.37 (1.14–1.65)**	12	74.7 (44.7–91.5)	1.05 (0.86–1.28)	12	82.6 (33.4–97.8)	1.05 (0.79–1.39)
**Location of prenatal care**	p*** = 0.002			p*** = 0.0003			p*** = 0.013	
Public	974	67.1 (62.9–71.1)	Ref.	1,038	71.5 (67.2–75.5)	Ref.	1,155	79.8 (75.9–83.3)	Ref.
Private	337	80.8 (74.3–85.9)	**1.20 (1.09–1.32)**	354	84.4 (78.4–89.0)	**1.17 (1.08–1.28)**	374	88.6 (82.2–92.9)	**1.10 (1.02–1.19)**
Others	9	63.6 (24.7–90.3)	0.94 (0.51–1.74)	11	91.2 (71.1–97.7)	**1.27 (1.10–1.46)**	14	96.9 (79.5–99.6)	**1.21 (1.12–1.31)**

Note: *sample n; ** population prevalence; *** Pearson’s chi-square test; 95%CI: 95% Confidence Interval; uPR: unadjusted Prevalence Ratio.

The prevalence of having received counseling on breastfeeding during pregnancy was 82.4%; however, women with low levels of education, black and brown women and who received prenatal care in the public health system received counseling in less than 80% of cases.

## DISCUSSION

The results reveal inequalities in the receipt of counseling, especially among Brazilian women who live in less economically developed regions of the country, who have brown or black skin color, who have low levels of education and who receive prenatal care in the public health system, despite the high prevalence of counseling received during prenatal care. It is worth noting that inequalities can constitute vulnerabilities, in the sense of greater susceptibility to potential harm during pregnancy and postpartum, especially for adolescent women, primigravidae, those with nutritional deficiencies, low levels of education, migrants and refugees^(5)^.

The finding of this study, of the lower prevalence of counseling received by these women, supports data presented by a cross-sectional population-based study that concluded that, in Brazil, being black and occupying unfavorable social positions entail disadvantages in accessing prenatal care that meets the Ministry of Health criteria^(17)^. Furthermore, black postpartum women have a higher risk of having inadequate prenatal care, lack of connection to the maternity hospital, absence of a companion, pilgrimage to birth and less local anesthesia for episiotomy, revealing an internalized racism in healthcare professionals who consider black women as more resistant to pain^(18)^.

It is recognized that black Brazilian women face overlapping conditions of vulnerability, since they experience experiences of social exclusion related to structural racism and gender inequalities, requiring strategic actions with an intersectional approach to address these conditions and ensure equal quality in prenatal care^(19,20)^. Inequalities in the provision of counseling for women are also associated with the region, which may be linked to prenatal coverage, since, in the North and Northeast regions of the country, there is low coverage of appointments for pregnant women, as identified in a previous study^(9)^.

When analyzing the topics of the guidelines received, it is observed that the lowest prevalences are related to guidelines on signs of labor and signs of risk during pregnancy. Specific guidelines on pregnancy, childbirth and the postpartum period can contribute to reducing the length of hospital stay for women in labor and, as a consequence, reduce the risks of unnecessary interventions during labor and delivery, ensuring more favorable obstetric and perinatal outcomes^(21)^. The need to address specific guidelines on the mode of delivery, such as quaternary prevention and for women’s informed choice, is highlighted, given contexts of high cesarean section rates^(22)^, as in Brazil.

Regarding counseling on referral maternity, there was an increase in prevalence from 2013 to 2019, showing an increase in the receipt of this counseling. Counseling on referral maternity aims to facilitate users’ access to the healthcare service at the time of delivery or in the event of complications, thus seeking to reduce the pregnant woman’s pilgrimage through the network, which still has high rates in the country^(23,24)^.

The connection between pregnant women and maternity was recommended with the implementation of the Stork Network, and should occur from the first prenatal appointment, favoring referral and counter-referral as well as responsible discharge and continuity of care after birth^(7)^. More recently, the Alyne Network, established by the Ministry of Health, restructured the Stork Network with the aim of consolidating a model of humanized and comprehensive care for pregnant women, women in labor, women in the postpartum period and children^(25)^, with a view to eliminating pilgrimages for childbirth and reducing maternal deaths, including the highlighted component of racial inequality.

Guidelines related to lifestyle habits, such as maintaining a healthy diet, not smoking and not drinking, showed high prevalence. Adherence to a healthy lifestyle during pregnancy is associated with a reduced risk of diseases, such as gestational diabetes and the risk of changes in children’s psychomotor development and, therefore, should be considered as an effective strategy in preventing complications^(26)^.

It was also observed that, in the 2013 PNS questionnaire, counseling on the risk of high blood pressure and risk of high blood sugar had a prevalence above 90%, being two of the most frequent guidelines in this study. However, these guidelines were offered mainly to those women diagnosed with these comorbidities, evidencing a biological approach in prenatal care, as evidenced in previous studies^(7)^. Therefore, it is necessary that the information offered to pregnant women is not only for risk control, but that it facilitates health education as an instrument for the dialogical construction of knowledge, encouraging autonomy, popular participation and women’s leading role in their own care^(27)^.

Regarding care variables, it was observed that being treated in the public health system is associated with a lower prevalence of receiving counseling. This finding shows that this variable may have functioned more as a social marker than as a care marker, considering that women assisted in this service present greater social vulnerability^(16)^ and, as previously presented, pregnant women exposed to conditions of inequality are subject to receiving prenatal counseling less frequently.

Thus, our findings demonstrate inequities, as women with some social vulnerability are receiving less care or assistance less frequently than others, which points to the need to develop strategies aimed at less favored populations, seeking to facilitate contact with healthcare services and early initiation of prenatal care, as well as investing in healthcare professionals’ qualification to better understand their role in offering equitable care, committed to tackling inequalities and social transformation. The Alyne Network anticipates these needs, and this study can become a reference point for future assessments with the implementation of new strategies. In this regard, it is also necessary to highlight the importance of nursing professionals, in the international and national scenario, whose scope of action includes interpersonal and educational relationships in health beyond disease control, encouraging the promotion of healthy lifestyles, the socialization of knowledge and the formation of critical and autonomous subjects in self-care^(27)^. Nurses are responsible for prenatal care in most countries, including nurses-midwives, and therefore expanding their skills in health promotion and health behavior change is essential^(27)^.

Therefore, the identification of conditions of inequality in access to healthcare, as surveyed in this research, contributes to informing specific interventions and public policies aimed at the constant improvement of the care offered and, consequently, the reduction of maternal and neonatal morbidity and mortality^(28)^. Despite advances in obstetric care in the country and the evidence from this study of the high prevalence of counseling during prenatal care, political changes such as the recent interruption of the Stork Network for the Maternal and Child Care Network (Rami), with a single-professional, doctor-centered focus and disconnected from other spheres of government^(29)^, in a conservative political context that interrupted several policies to guarantee women’s rights, together with SUS defunding^(12,30)^ and the pandemic, may have contributed to the deepening of these inequalities. This reality may even be experienced by other countries. Therefore, future studies will be necessary for the continuous monitoring of this assistance in the country, especially with the establishment of a new government strategy, with a view to addressing these persistent and worsening setbacks.

Concerning study limitations, the possibility of memory bias in the data collected is recognized, since, in PNS interviews, information about childbirth in the last two years was asked. Another limitation is the lack of representativeness of the sample of the Asian and indigenous population, whose data should be interpreted with caution. Furthermore, it was not possible to assess progress or setbacks regarding the receipt of most of the guidelines addressed in the 2013 PNS, with the exception of counseling on reference maternity, since other guidelines were not included in the 2019 PNS questionnaire. In addition to not allowing the monitoring of these indicators, the non-inclusion of guidelines in the questionnaire reinforces care that is exclusively focused on techniques and biological aspects. It also points to the devaluation of health education as a soft technology of care, portrayed in this study as a guiding axis of professional practice for health promotion and recovery, and the emancipation of pregnant women in the care process.

## CONCLUSION

The results of this study show that prenatal counseling is being provided by professionals, with emphasis on counseling on reference maternity, which showed a significant increase in prevalence from 2013 to 2019. However, they point to inequalities and inequities in this assistance, such as less counseling received by women with low levels of education, brown and black women, who live in the Northeast region, who underwent prenatal care in the public health system and, mainly, with a lower prevalence of counseling regarding the pregnancy-puerperal cycle specificities. Therefore, it is believed that studies like this, which assess the counseling that is being provided to pregnant women during prenatal care at a national level and which cover a greater number of guidelines, can contribute to a better assessment of the reality of this assistance. Added to this is the importance of strengthening the participation of nurses in offering comprehensive care for women’s health, with a focus on health promotion and greater commitment to addressing conditions of inequality in the provision of counseling during prenatal care. This study may become a reference point for future assessments, given the political-programmatic setbacks in the area of women’s health in the country, which may also be the reality in other countries, given the growing conservatism, pandemics, wars and situations that put women’s health and the guarantee of their rights at greater risk.
