# Supplementary material for: Counseling received in prenatal care by Brazilian women
Source: Rev Esc Enferm USP. 2025 Apr 25;59:e20240198. doi: 10.1590/1980-220X-REEUSP-2024-0198en (PMC12024353; doi:10.1590/1980-220X-REEUSP-2024-0198en)
Supplement: Translation

# Translation: Orientações recebidas no cuidado pré-natal pelas mulheres brasileiras

**DOI:** 10.1590/1980-220X-REEUSP-2024-0198pt

**Authors:** Ana Clara Alves Tomé de Souza, Marina Garcia Avelar Rocha, Kênia Lara Silva, Rafaela Siqueira Costa Schreck, Eunice Francisca Martins, Nágela Cristine Pinheiro Santos, Mariana Santos Felisbino-Mendes

**Affiliations:** 1 Universidade Federal de Minas Gerais, Escola de Enfermagem, Belo Horizonte, MG, Brazil.; 2 Universidade Federal de Minas Gerais, Escola de Enfermagem, Departamento de Enfermagem Aplicada, Belo Horizonte, MG, Brazil.; 3 Universidade Federal de Minas Gerais, Escola de Enfermagem, Departamento de Enfermagem Materno-Infantil e Saúde Pública, Belo Horizonte, MG, Brazil

**Keywords:** Educação em Saúde, Cuidado Pré-Natal, Fatores Socioeconômicos, Saúde da Mulher, Desigualdades de Saúde

## Abstract

**Objetivo:**

Analisar as orientações recebidas durante o pré-natal pelas mulheres brasileiras e sua associação com as características sociodemográficas e assistenciais.

**Método:**

Estudo transversal, que utilizou dados provenientes das edições da Pesquisa Nacional de Saúde de 2013 e 2019. Foram estudadas mulheres de 18 a 49 anos que realizaram pré-natal em até dois anos antes do inquérito (1.851 em 2013 e 2.819 em 2019). Estimaram-se as prevalências com seus Intervalos de Confiança de 95% das diferentes orientações. Em seguida, estimaram-se prevalências, Razão de Prevalência não ajustada e Intervalo de 95% de Confiança segundo variáveis sociodemográficas e assistenciais.

**Resultados:**

Os resultados revelam desigualdades nas orientações recebidas durante o pré-natal, principalmente entre mulheres que vivem em regiões menos desenvolvidas do país, de cor da pele parda/negra, de baixa escolaridade e que realizam o pré-natal no sistema público de saúde.

**Conclusão:**

As orientações são realizadas pelos profissionais, com destaque para orientação sobre maternidade de referência, mas há iniquidades na oferta dessas orientações.

## INTRODUÇÃO

A atenção obstétrica deve ser pautada no cuidado interprofissional prestado à mulher durante o pré-natal, parto e puerpério. A mulher necessita ser integrada como protagonista desse cuidado e das suas escolhas, com acesso às ações de rastreio, diagnóstico e prevenção de agravos, acrescidas de informações sobre os aspectos fisiológicos e comportamentais, baseadas em evidências científicas, além de apoio em relação às questões sociais e emocionais^(1)^. Assim, a utilização de tecnologias leves, como escuta qualificada e humanização do atendimento^(2)^, que são inerentes às boas práticas obstétricas, pode contribuir para que a mulher vivencie de forma positiva a experiência da maternidade, a qual envolve autoestima, competência e autonomia, advindas da segurança, respeito e dignidade, por meio da comunicação, acolhimento e educação em saúde^(1)^.

Nesse sentido, considera-se a educação em saúde como uma prática social e o eixo norteador da assistência pré-natal^(3)^. A partir dessa perspectiva, compreende-se que todas as consultas são oportunidades para realizar intervenções educativas individuais ou coletivas, as quais têm sido também recomendadas para reparação de vulnerabilidades durante a gestação^(4,5)^. Para isso, a educação em saúde assume um compromisso humanista e transformador que, sob uma concepção freireana, deve ser sustentada pelo diálogo entre educador e educando, em um processo multidimensional que considere aspectos culturais, econômicos e sociais^(6)^. Entretanto, estudos têm identificado que os profissionais de saúde não têm realizado ou têm realizado, parcialmente, a educação em saúde no atendimento às gestantes^(7,8)^. Ao avaliar a qualidade da atenção pré-natal na rede básica de saúde do Brasil, constatou-se que apenas 60% das gestantes receberam orientações referentes à assistência ao pré-natal, tais como aleitamento materno exclusivo, alimentação e ganho de peso, cuidados com o recém-nascido e importância do papanicolau^(9)^. Outros achados também identificaram acesso limitado a orientações para o parto^(10)^ e sobre a maternidade de referência^(11)^, apontando um déficit em relação à educação em saúde realizada no pré-natal. Quando identificada a completude de ações preconizadas e orientações no pré-natal, estas foram significativamente maiores para gestantes com mais idade e maior renda, e em municípios de maior porte^(9)^. Tal achado revela que persistem desigualdades sociais e individuais na qualidade do pré-natal ofertado às mulheres no país, comprometendo a garantia do princípio da equidade no âmbito do Sistema Único de Saúde (SUS). Ademais, persistem modelos assistenciais focados na doença e procedimentos técnicos, em detrimento de um modelo de cuidado pautado na integralidade à saúde com ações de educação em saúde^(12)^.

Dessa forma, as orientações fornecidas às gestantes são indicadores que podem avaliar a qualidade da assistência ao pré-natal, tendo em vista sua associação direta com a promoção da saúde. Entretanto, a maioria dos estudos encontrados na literatura avalia a qualidade da assistência pré-natal de modo local/regional e apenas por meio de marcadores de acesso e/ou marcadores clínicos^(11,13-16)^. Quando avaliadas, as orientações são contempladas em menor número, muitas vezes não identificando os subgrupos mais afetados com a falta dessa assistência, fato que também pode ser observado nos estudos nacionais^(12,15)^.

Diante do exposto, o estudo teve como objeto as orientações fornecidas às gestantes durante a consulta de pré-natal. Estudos desta natureza podem contribuir para uma melhor avaliação da realidade da assistência pré-natal em nível populacional, avaliando outros elementos além dos marcadores de acesso. Assim, o objetivo deste estudo foi analisar as orientações recebidas durante o pré-natal pelas mulheres brasileiras e sua associação com as características sociodemográficas e assistenciais.

## MÉTODO

### Tipo do Estudo

Trata-se de estudo transversal com dados da Pesquisa Nacional de Saúde (PNS), edições de 2013 e de 2019, que é inquérito brasileiro cujo objetivo principal é caracterizar a situação de saúde e os estilos de vida da população brasileira.

### Definição da Amostra

O processo de amostragem da PNS foi realizado em três estágios: no primeiro, selecionaram-se os setores censitários; no segundo, selecionaram-se os domicílios; e no terceiro, para cada domicílio, foi selecionado um morador de 18 anos ou mais de idade, em 2013, e 15 anos ou mais de idade, em 2019, todos por amostragem aleatória simples. O questionário da PNS, em ambas as edições, foi dividido em módulos que abrangem as características do domicílio, de todos os moradores e do morador adulto selecionado. No módulo S, foi investigado o atendimento pré-natal entre as mulheres que tiveram parto no período de 28/07/2011 a 27/07/2013, na edição de 2013, e no período igual ou posterior a 28/07/2017, na edição de 2019, ou seja, nos últimos dois anos antes dos inquéritos, considerando apenas o último parto para responder às questões. Entre as mulheres que responderam ao módulo S dos questionários, uma subamostra foi definida, sendo incluídas apenas as mulheres de 18 a 49 anos que realizaram pré-natal na última gestação, totalizando 1.851 mulheres disponíveis para o estudo, em 2013, e 2.819 mulheres, em 2019.

### Coleta de Dados

Neste estudo, foram analisadas dez perguntas específicas referentes às orientações fornecidas durante a assistência ao pré-natal, a seguir: durante as consultas de pré-natal, a Sra. recebeu alguma das seguintes orientações: não faltar às consultas, manter uma alimentação saudável, não fumar, não beber, sobre aleitamento materno, sinais de risco na gravidez, e sinais de trabalho de parto, riscos de pressão arterial elevada, risco do açúcar elevado no sangue e em qual serviço de saúde deveria ir no momento do parto?

Entretanto, observou-se que, comparado ao ano de 2013, apenas uma das orientações analisadas anteriormente foi mantida em 2019, que corresponde à orientação sobre qual serviço de saúde de referência que a mulher deveria ir no momento do parto. As demais orientações não foram investigadas, prejudicando o monitoramento desses indicadores. Consideraram-se as orientações como variáveis dependentes, e como independentes, utilizaram-se as seguintes variáveis sociodemográficas: faixa etária (18–29 anos, 30–39 anos e 40–49 anos); região (Norte, Nordeste, Centro-Oeste, Sudeste e Sul); escolaridade, em anos (0–8 anos, 9–11 anos ou 12 ou mais); cor da pele (branca, amarela, parda, preta e indígena); e local de atendimento do pré-natal (sistema de saúde público, privado ou outro local).

### Análise e Coleta dos Dados

Primeiramente, estimaram-se as prevalências com seus Intervalos de 95% de Confiança das diferentes orientações recebidas pelas mulheres em 2013 e 2019. Em seguida, estimaram-se essas prevalências segundo as variáveis sociodemográficas e assistenciais. Utilizou-se o teste qui-quadrado de Pearson para avaliar diferenças estatísticas entre as prevalências. Finalmente, estimaram-se a Razão de Prevalência não ajustada (RPna) e o Intervalo de 95% de Confiança de ter recebido orientações para os diferentes grupos das variáveis independentes somente para os fatores que na análise descritiva apresentaram um p < 0,2. Os dados foram analisados com uso do programa estatístico Stata, versão 14.2, utilizando o módulo *survey* para obtenção de estimativas populacionais, considerando estrato, conglomerado e peso dos indivíduos da amostra complexa.

### Aspectos Éticos

Este estudo utiliza dados secundários da PNS disponíveis publicamente em sites oficiais do Ministério da Saúde do Brasil ou Instituto Brasileiro de Geografia e Estatística, sendo dispensado de análise em Comitê de Ética em Pesquisa, conforme a Resolução no 510/2016 do Conselho Nacional de Saúde. A PNS recebeu aprovação pela Comissão Nacional de Ética em Pesquisa em julho de 2013 e em agosto de 2019.

## RESULTADOS

Observa-se que tanto em 2013 quanto em 2019, respectivamente, a maioria das mulheres que tiveram parto nos dois últimos anos antes dos inquéritos tinha idade entre 18–29 anos (61,2%/52,7%), residia na região Sudeste (38,1%/36,9%), seguida da Nordeste (29,1%/29,5%), tinha escolaridade entre 9–11 anos (47,2%/8,6%), autodeclarou-se como parda (50,0%/51,7%) e utilizou o serviço público de saúde para o atendimento pré-natal (71,9%/70%) (Tabela 1).

**Tabela 1:** Prevalência e Intervalos de Confiança de 95% das características sociodemográficas e assistenciais das mulheres brasileiras que realizaram pré-natal na Pesquisa Nacional de Saúde – Brasil, 2013 e 2019.

Variáveis sociodemográficas e assistenciais	2013		2019	
	n*	%** (IC95%)	n*	%** (IC95%)
**Faixa etária (anos)**				
18–29	1,120	61,2 (57,4–64,9)	1,456	52,7 (49,3–55,9)
30–39	662	35,2 (31,7–38,9)	1,124	41,5 (38,3–44,8)
40–49	69	3,6 (2,5–5,1)	149	5,7 (4,4–7,3)
**Região**				
Centro–Oeste	232	8,2 (7,0–9,6)	344	9,2 (7,9–10,8)
Norte	517	9,9 (8,5–11,5)	687	11,6 (10,3–13,1)
Nordeste	565	29,1 (25,9–32,7)	1,041	29,5 (26,9–32,2)
Sudeste	345	38,1 (34,1–42,3)	476	36,9 (33,4–40,5)
Sul	192	14,6 (12,1–17,5)	271	12,5 (10,8–14,5)
**Escolaridade (anos)**				
0–8	707	36,7 (33,1–40,0)	900	27,6 (24,8–30,6)
9–11	830	47,2 (43,4–51,1)	1,310	48,6 (45,3–51,8)
12 ou mais	314	16,1 (13,5–19,1)	609	23,7 (21,0–26,6)
**Cor da pele/raça**				
Branca	593	39,9 (36,1–43,8)	802	35,1 (32,0–38,3)
Preta	167	8,8 (6,9–11,2)	293	11,8 (9,6–14,3)
Parda	1,052	50,0 (46,2–53,8)	1,665	51,7 (48,4–54,9)
Amarela	24	1,1 (0,006–2,1)	31	1,0 (0,6–1,5)
Indígena	15	0,1 (0,0006–0,6)	28	0,6 (0,3–1,0)
**Local de realização do pré–natal**				
**Público**	1,401	71,9 (68,0–75,5)	2,141	70,0 (66,7–73,1)
**Privado**	435	27,3 (23,8–31,2)	677	29,9 (26,8–33,2)
**Outros**	15	0,7 (0,3–1,5)	1	0,1 (0–1,0)

Nota: *n amostral; ** prevalência populacional; IC95%: Intervalo de 95% Confiança

Foi observado um aumento significativo da prevalência da orientação sobre a maternidade de referência de 2013 (74,9%) para 2019 (83,0%) (Tabela 2). Entretanto, observaram-se desigualdades na oferta dessa orientação nos dois períodos, uma vez que as mulheres da faixa etária de 18–29 anos, que viviam nas regiões Norte e Nordeste, com baixa escolaridade, indígenas, pretas e pardas, que realizaram o pré-natal no sistema de saúde público, apresentaram menor prevalência de serem orientadas.

**Tabela 2:** Prevalência, Razão de Prevalência não ajustada e Intervalos de Confiança de 95% das orientações gerais recebidas durante o cuidado pré-natal das mulheres brasileiras com parto nos últimos dois anos antes dos inquéritos na Pesquisa Nacional de Saúde – Brasil, 2013 e 2019.

Variáveis sociodemográficas e assistenciais	Maternidad de referencia/2013			Maternidad de referencia/2019			Não faltar às consultas		
	n*	%** (IC95%)	RPna (IC95%)	n*	%**(IC95%)	RPna (IC95%)	n*	%**(IC95%)	RPna (IC95%)
**Recebeu orientação**	1,362	74,9 (71,4–78,1)	-	2,244	83,0 (80,7–85,1)	-	1,680	88,9 (85,8–91,4)	-
**Faixa etária (anos)**		p*** = 0,011			p*** = 0,0006			p*** = 0,338	
18–29	807	71,1 (66,3–75,5)	Ref.	1,113	79,0 (75,3–82,3)	Ref.	1,018	87,5 (83,1–90,9)	-
30–39	505	81,1 (76,0–85,4)	**1,14 (1,04–1,24)**	939	88,2 (84,9–90,8)	**1,08 (1,02–1,15)**	598	91,5 (87,4–94,3)	-
40–49	50	77,0 (58,3–88,9)	1,08 (0,87–1,33)	124	85,5 (75,0–92,1)	1,15 (0,99–1,34)	64	87,0 (63,7–96,3)	-
**Região**		p*** < 0,0001			p***< 0,0001			p***= 0,530	
Centro–Oeste	168	71,2 (62,9–78,2)	Ref.	298	87,9 (82,3–91,8)	Ref.	203	86,0 (79,5–90,7)	-
Norte	380	72,6 (64,9–79,1)	1,02 (0,88–1,18)	504	72,4 (66,6–77,6)	0,88 (0,80–0,98)	478	93,4 (89,1–96,1)	-
Nordeste	368	61,0 (53,9–67,7)	0,85 (0,73–1,00)	781	75,9 (71,6–79,7)	0,87 (0,81–0,94)	516	90,0 (83,6–94,3)	-
Sudeste	279	83,6 (77,5–88,3)	**1,17 (1,03–1,33)**	410	87,7 (82,6–91,5)	1,01 (0,93–1,08)	309	87,3 (81,0–91,7)	-
Sul	167	83,3 (74,2–89,7)	**1,17 (1,01–1,34)**	251	92,3 (87,6–95,4)	1,09 (1,00–1,18)	174	89,0 (79,8–94,3)	-
**Escolaridade (anos)**		p*** = 0,051			p*** = 0,0002			p*** = 0,656	
0–8	491	69,4 (63,7–74,6)	Ref.	675	78,0 (73,6–81,9)	Ref.	652	90,4 (85,1–93,9)	-
9–11	620	77,7 (72,6–82,0)	**1,11 (1,01–1,23)**	1,026	81,7 (78,0–84,9)	1,02 (0,95–1,10)	754	87,7 (82,6–91,5)	-
12 ou +	251	79,0 (69,6–86,0)	1,13 (1,00–1,29)	543	91,5 (87,0–94,5)	**1,11 (1,03–1,19)**	274	88,8 (82,0–93,2)	-
**Cor da pele/raça**		p*** < 0,0001			p*** = 0,008			p*** = 0,735	
Branca	475	84,0 (79,2–87,8)	Ref.	683	88,7 (84,8–91,7)	Ref.	534	89,8 (85,0–93,2)	-
Preta	116	72,6 (61,9–81,2)	1,09 (1,00–1,19)	238	84,3 (75,1–90,6)	1,04 (0,97–1,12)	156	87,6 (72,9–94,9)	-
Parda	743	68,5 (63,3–73,3)	**1,13 (1,07 –1,19)**	1,270	80,4 (77,2–83,3)	**1,07 (1,03–1,12)**	952	88,3 (83,9–91,6)	-
Amarela	17	79,2 (52,9–92,8)	1,04 (0,88–1,23)	25	86,0 (64,1–95,4)	1,02 (0,90–1,17)	24	100,0	-
Indígena	11	54,3 (18,0–86,6)	1,26 (0,94–1,68)	25	98,0 (91,9–99,5)	**0,92 (0,88–0,96)**	14	97,3 (78,9–99,7)	-
**Local de realização do pré–natal**	p*** < 0,0001			p*** = 0,002			p*** = 0,137	
Público	993	69,4 (65,0–73,5)	Ref.	1,619	79,9 (77,1–82,4)	Ref.	1,286	89,5 (85,9–92,3)	-
Privado	356	88,7 (84,0–92,0)	**1,27 (1,18–1,38)**	614	90,3 (85,7–93,6)	**1,07 (1,01–1,13)**	384	88,0 (81,2–92,5)	-
Outros	13	95,8 (80,7–99,2)	**1,38 (1,25–1,51)**	01	100,0	**1,18 (1,14–1,22)**	10	60,9 (23,5–88,8)	-

Nota: *n amostral; ** prevalência populacional; *** teste qui-quadrado de Pearson; IC95%: Intervalo de Confiança de 95%; RPna: Razão de Prevalência não ajustada.

A orientação sobre não faltar às consultas apresentou prevalência elevada (88,9%). Não houve associação entre as variáveis sociodemográficas e assistenciais com essa orientação. Quanto às orientações relacionadas aos hábitos de vida, como manter uma alimentação saudável, não fumar e não beber, independentemente das variáveis sociodemográficas e assistenciais, a prevalência de as ter recebido foi superior a 80%, e a orientação sobre alimentação saudável foi a mais frequente entre todas essas (96,8%) (Tabela 3).

**Tabela 3:** Prevalência, Razão de Prevalência não ajustada e Intervalos de Confiança de 95% de orientações recebidas sobre os hábitos de vida durante o cuidado pré-natal das mulheres brasileiras com parto nos últimos dois anos antes dos inquéritos na Pesquisa Nacional de Saúde – Brasil, 2013 e 2019.

Variáveis sociodemográficas e assistenciais	Alimentação saudável			Não fumar			Não beber		
	n*	%** (IC95%)	RPna (IC95%)	n*	%**(IC95%)	RPna (IC95%)	n*	%**(IC95%)	RPna (IC95%)
**Recebeu orientação**	1,792	96,8 (95,2–97,9)	-	1,684	89,1 (86,0–91,6)	-	1,687	89,9 (87,1–92,2)	-
**Faixa etária (anos)**		p*** = 0,491			p*** = 0,824			p*** = 0,291	
18–29	1,080	96,4 (94,0–97,8)	-	1,015	88,6 (84,7–91,6)	-	1,019	89,2 (85,4–92,2)	Ref.
30–39	643	97,2 (94,8–98,5)	-	603	89,7 (83,7–93,7)	-	602	90,3 (85,3–93,7)	1,02 (0,96–1,09)
40–49	69	100	-	66	92,8 (66,9–98,8)	-	66	97,5 (91,1–99,3)	1,00 (0,91–1,10)
**Região**		p*** = 0,361			p*** = 0,153			p*** = 0,559	
Centro–Oeste	226	96,4 (91,9–98,4)	-	211	88,6 (80,1–93,7)	Ref.	210	88,6 (80,2–93,7)	-
Norte	501	98,0 (96,3–99,0)	-	482	92,9 (86,5–96,3)	1,04 (0,96–1,14)	482	93,0 (86,9–96,4)	-
Nordeste	548	98,0 (96,2–99,0)	-	516	92,4 (86,5–95,8)	1,04 (0,95–1,14)	513	91,5 (85,7–95,1)	-
Sudeste	335	96,4 (92,6–98,3)	-	299	85,4 (79,0–90,1)	0,96 (0,87–1,06)	302	86,7 (82,1–91,7)	-
Sul	182	94,8 (87,6–97,9)	-	176	89,9 (81,5–94,8)	1,01 (0,91–1,12)	180	91,0 (81,8–95,8)	-
**Escolaridade (anos)**		p*** = 0,072			p*** = 0,970			p*** = 0,706	
0–8	685	97,1 (94,8–98,4)	Ref.	646	88,8 (93,5–92,5)	-	648	88,7 (83,4–92,5)	-
9–11	798	95,8 (92,6–97,6)	0,98 (0,95–1,01)	754	89,5 (84,8–92,8)	-	755	91,0 (87,3–93,7)	-
12 ou +	309	99,2 (97,5–99,8)	1,02 (1,00–1,04)	284	88,9 (79,8–94,2)	-	284	89,3 (80,5–94,4)	-
**Cor da pele/raça**		p*** = 0,890			p*** = 0,494			p*** = 0,258	
Branca	574	96,3 (92,7–98,2)	-	535	90,1 (85,9–93,1)	-	541	91,9 (88,0–94,6)	-
Preta	162	97,1 (92,1–99,0)	-	151	90,9 (79,7–96,2)	-	149	90,3 (79,4–95,8)	-
Parda	1,018	97,0 (94,8–98,2)	-	960	87,3 (82,0–91,1)	-	959	87,6 (83,0–91,1)	-
Amarela	24	100,0	-	24	100,0	-	24	100,0	-
Indígena	14	97,3 (78,9–99,7)	-	14	97,3 (78,9–99,7)	-	14	97,3 (78,9–99,7)	-
**Local de realização do pré–natal**	p*** = 0,105			p*** = 0,542			p*** = 0,116	
Público	1,352	96,2 (94,0–97,6)	Ref.	1,278	89,0 (85,3–91,8)	-	1,277	88,8 (85,1–91,7)	Ref.
Privado	426	98,2 (95,8–99,3)	1,02 (0,99–1,04)	392	89,2 (82,4–93,6)	-	396	92,5 (88,0–95,3)	1,04 (0,98–1,09)
Outros	18	99,4 (95,3–99,9)	1,03 (1,01–1,05)	14	99,4 (95,3–99,9)	-	14	99,4 (95,3–99,9)	1,11 (1,07–1,16)

Nota: *n amostral; ** prevalência populacional; *** teste qui-quadrado de Pearson; IC95%: Intervalo de 95% Confiança; RPna: Razão de Prevalência não ajustada.

Observa-se ainda que a prevalência das orientações sobre risco de pressão arterial elevada foi de 91,2% e risco do açúcar elevado no sangue de 92,9%, demonstrando serem frequentes para a maioria das mulheres que apresentaram pressão arterial elevada e glicemia alterada durante a gestação, uma vez que só foi perguntado sobre essas orientações para quem apresentava essas alterações (Tabela 4). Não houve associação entre as variáveis sociodemográficas e assistenciais e receber as orientações sobres os hábitos de vida e riscos no período gestacional retratados acima, com exceção da orientação sobre risco de açúcar elevado no sangue, no qual o atendimento pré-natal realizado no sistema de saúde público está associado a menor prevalência de receber tal orientação (Tabelas 3 e 4).

**Tabela 4:** Prevalência, Razão de Prevalência não ajustada e Intervalos de Confiança de 95% de orientações recebidas quanto aos riscos durante a gestação mediante elevação de pressão arterial e glicemia das mulheres brasileiras com parto nos últimos dois anos antes dos inquéritos na Pesquisa Nacional de Saúde – Brasil, 2013 e 2019.

Variáveis sociodemográficas e assistenciais	Risco de pressão arterial elevada			Risco de açúcar elevado no sangue		
	n*	%** (IC95%)	RPna (IC95%)	n*	%**(IC95%)	RPna (IC95%)
**Recebeu orientação**	347	91,2 (85,7–94,7)	-	89	92,9 (86,0–96,5)	-
**Faixa etária (anos)**		p*** = 0,719			p*** = 0,671	
18–29	191	90,0 (82,0–94,7)	-	42	90,6 (78,5–96,2)	-
30–39	134	92,6 (83,0–96,9)	-	40	94,1 (80,9–98,4)	-
40–49	22	92,7 (69,8–98,6)	-	7	100,0	-
**Região**		p*** = 0,610			p*** = 0,177	
Centro–Oeste	50	94,1 (82,1–98,3)	-	8	77,0 (38,0–94,8)	Ref.
Norte	102	89,9 (81,7–94,7)	-	27	87,7 (69,9–95,7)	1,14 (0,75–1,72)
Nordeste	106	94,2 (88,7–97,1)	-	18	92,1 (68,2–98,5)	1,19 (0,79–1,80)
Sudeste	51	90,6 (78,3–96,3)	-	19	95,7 (82,2–99,1)	1,24 (0,83–1,84)
Sul	38	85,7 (58,3–96,5)	-	17	98,9 (91,7–99,8)	1,28 (0,86–7,89)
**Escolaridade (anos)**		p*** = 0,921			p*** = 0,553	
0–8	145	90,0 (79,7–95,4)	-	29	91,8 (76,7–97,4)	-
9–11	150	92,0 (83,6–96,3)	-	36	91,3 (79,0–96,7)	-
12 ou +	52	91,1 (70,5–97,8)	-	24	100,0	-
**Cor da pele/raça**		p*** = 0,473			p*** = 0,936	
Branca	103	87,6 (74,7–94–4)	-	27	92,5 (73,1–98,2)	-
Preta	31	93,6 (80,8–98,1)	-	4	88,8 (45,0–98,7)	-
Parda	203	92,9 (85,5–96,6)	-	54	92,7 (83,7–96,9)	-
Amarela	4	100,0	-	3	100,0	-
Indígena	6	70,7 (14,9–97,1)	-	1	100,0	-
**Local de realização do pré–natal**		p*** = 0,092			p***<0,0001	
Público	275	89,4 (82,5–93,7)	Ref.	61	91,0 (81,4–95,9)	Ref.
Privado	71	96,6 (82,9–99,4)	1,08 (0,99–1,17)	28	99,0 (92,6–99,9)	1,17 (1,08–1,28)
Outros	01	41,1 (4,2–91,8)	0,46 (0,08–2,35)	0	0	-

Nota: *n amostral; ** prevalência populacional; *** teste qui-quadrado de Pearson; IC95%: Intervalo de 95% Confiança; RPna: Razão de Prevalência não ajustada.

Em relação a todas as orientações estudadas, observou-se menor prevalência das orientações específicas do ciclo gravídico puerperal, como sinais de trabalho de parto (70,8%) e sinais de risco na gravidez (75,2%), sendo a orientação sobre sinais de trabalho de parto a menos prevalente (Tabelas 2 e 5). Ainda, observa-se que alguns subgrupos populacionais receberam menos orientações específicas da gestação em detrimento de outros, como as mulheres pretas, pardas, amarelas e indígenas, com escolaridade entre 0–8 anos, que residiam na região Nordeste e realizaram o pré-natal no sistema de saúde público (Tabela 5), assim como foi observado sobre a maternidade de referência. Destaca-se que, quando relacionadas tais orientações à escolaridade, a prevalência é 15%, menor entre mulheres de baixa escolaridade comparadas com as de alta escolaridade (Tabela 5).

**Tabela 5:** Prevalência, Razão de Prevalência não ajustada e Intervalos de Confiança de 95% de orientações específicas do ciclo gravídico-puerperal recebidas durante o cuidado pré-natal das mulheres brasileiras com parto até dois anos antes do inquérito na Pesquisa Nacional de Saúde – Brasil, 2013 e 2019.

Variáveis sociodemográficas e assistenciais	Sinais de trabalho de parto			Sinais de risco na gravidez			Aleitamento materno		
	n*	%** (IC95%)	RPna (IC95%)	n*	%**(IC95%)	RPna (IC95%)	n*	%**(IC95%)	RPna (IC95%)
**Recebeu orientação**	1,320	70,8 (67,3–74,2)	-	1,403	75,2 (71,7–78,4)	-	1,543	82,4 (79,7–85,2)	-
**Faixa etária (anos)**		p*** = 0,048			p*** = 0,019			p*** = 0,123	
18–29	796	68,7 (63,9–73,2)	Ref.	831	72,0 (67,1–76,3)	Ref.	932	80,0 (75,5–83,8)	Ref.
30–39	476	75,9 (70,4–80,6)	1,10 (1,00–1,22)	517	79,9 (74,6–84,4)	1,11 (1,01–1,21)	553	86,4 (81,6–90,2)	1,08 (1,00–1,16)
40–49	48	57,8 (38,8–74,7)	0,84 (0,60–1,17)	55	84,2 (70,8–92,2)	1,17 (1,01–1,34)	58	82,4 (79,0–85,2)	1,03 (0,85–1,25)
**Região**		p*** = 0,028			p*** = 0,088			p*** = 0,288	
Centro–Oeste	155	67,4 (59,9–74,3)	Ref.	175	74,1 (65,9–80,9)	Ref.	184	80,2 (73,0–85,9)	-
Norte	360	68,4 (60,6–75,4)	1,01 (0,87–1,18)	392	75,5 (67,8–81,9)	1,01 (0,88–1,17)	422	78,7 (69,6–85,6)	-
Nordeste	393	64,2 (57,5–70,5)	0,95 (0,82–1,10)	411	68,8 (62,0–74,8)	0,92 (0,90–1,06)	469	78,8 (72,4–84,1)	-
Sudeste	261	74,7 (67,9–80,4)	1,10 (0,96–1,26)	270	78,5 (71,9–83,9)	1,05 (0,93–1,20)	295	85,1 (78,9–89,7)	-
Sul	151	77,5 (69,1–84,2)	1,14 (0,99–1,32)	155	79,6 (70,3–86,6)	1,07 (0,92–1,24)	173	85,9 (76,5–92,0)	-
**Escolaridade (anos)**		p*** < 0,0001			p*** = 0,029			p*** = 0,0002	
0–8	467	61,6 (55,7–67,1)	Ref.	514	69,9 (64,0–75,1)	Ref.	559	75,7 (69,8–80,7)	Ref.
9–11	616	74,2 (68,7–79,0)	**1,20 (1,07–1,35)**	635	77,0 (71,9–81,5)	1,10 (0,99–1,21)	710	84,2 (79,2–88,2)	**1,11 (1,01–1,21)**
12 ou +	237	82,0 (75,7–87,0)	**1,33 (1,18–1,49)**	254	81,9 (73,6–78,4)	1,17 (1,04–1,31)	274	92,1 (87,5–95,1)	**1,21 (1,12–1,32)**
**Cor da pele/raça**		p*** = 0,0001			p*** = 0,015			p*** = 0,012	
Branca	444	79,3 (74,2–83,6)	Ref.	467	80,9 (75,6–85,3)	Ref.	514	88,1 (83,3–91,7)	Ref.
Preta	115	60,8 (48,4–71,9)	**1,15 (1,05–1,27)**	123	65,4 (52,6–76,3)	**1,13 (1,02–1,25)**	140	75,2 (61,7–85,1)	1,12 (1,00–1,23)
Parda	736	67,0 (61,9–71,6)	**1,10 (1,04–1,16)**	782	72,5 (67,4–77,1)	**1,07 (1,01–1,13)**	857	79,3 (74,8–83,2)	**1,08 (1,03–1,13)**
Amarela	17	42,5 (17,9–71,6)	**1,30 (1,07–1,58)**	19	58,8 (28,2–77,1)	1,19 (0,95–1,49)	20	77,2 (48,7–92,4)	1,10 (0,91–1,32)
Indígena	8	34,8 (12,6–66,5)	**1,37 (1,14–1,65)**	12	74,7 (44,7–91,5)	1,05 (0,86–1,28)	12	82,6 (33,4–97,8)	1,05 (0,79–1,39)
**Local de realização do pré–natal**	p*** = 0,002			p*** = 0,0003			p*** = 0,013	
Público	974	67,1 (62,9–71,1)	Ref.	1,038	71,5 (67,2–75,5)	Ref.	1,155	79,8 (75,9–83,3)	Ref.
Privado	337	80,8 (74,3–85,9)	**1,20 (1,09–1,32)**	354	84,4 (78,4–89,0)	**1,17 (1,08–1,28)**	374	88,6 (82,2–92,9)	**1,10 (1,02–1,19)**
Outros	9	63,6 (24,7–90,3)	0,94 (0,51–1,74)	11	91,2 (71,1–97,7)	**1,27 (1,10–1,46)**	14	96,9 (79,5–99,6)	**1,21 (1,12–1,31)**

Nota: *n amostral; ** prevalência populacional; *** teste qui-quadrado de Pearson; IC95%: Intervalo de 95% Confiança; RPna: Razão de Prevalência não ajustada.

A prevalência de ter recebido orientação sobre o aleitamento materno na gestação foi de 82,4%, entretanto mulheres de baixa escolaridade, pretas e pardas e que realizaram o pré-natal no sistema de saúde público foram orientadas em menos de 80%.

## DISCUSSÃO

Os resultados revelam desigualdades no recebimento das orientações, principalmente entre as mulheres brasileiras que vivem em regiões economicamente menos desenvolvidas do país, de cor da pele parda e preta, com baixa escolaridade e que realizam o pré-natal no sistema público de saúde, apesar das elevadas prevalências das orientações recebidas durante o pré-natal. Destaca-se que as desigualdades podem se constituir em vulnerabilidades, no sentido de maior suscetibilidade a potenciais danos durante a gestação e pós-parto, especialmente para as mulheres adolescentes, primigestas, com deficiências nutricionais, baixa escolaridade, migrantes e refugiadas^(5)^.

O achado deste estudo, da menor prevalência das orientações recebidas por essas mulheres, corrobora dados apresentados por investigação transversal de base populacional que concluiu que, no Brasil, ser negra e ocupar lugares sociais desfavoráveis acarretam desvantagens no acesso a um pré-natal que atenda aos critérios estabelecidos pelo Ministério da Saúde^(17)^. Além disso, puérperas de cor preta possuem maior risco de terem um pré-natal inadequado, falta de vinculação à maternidade, ausência de acompanhante, peregrinação para o parto e menos anestesia local para a episiotomia, revelando um racismo internalizado nos profissionais de saúde que consideram as mulheres pretas como mais resistentes à dor^(18)^.

Reconhece-se que as mulheres negras brasileiras enfrentam condições sobrepostas de vulnerabilidades, uma vez que vivenciam experiências de exclusão social pertencentes ao racismo estrutural e às desigualdades de gênero, exigindo ações estratégicas de abordagem interseccional para enfrentamento dessas condições e garantia igualitária da qualidade na atenção ao pré-natal^(19,20)^. As desigualdades na oferta de orientações para mulheres também estão associadas à região, que podem estar interligadas à cobertura do pré-natal, pois, nas regiões Norte e Nordeste do país, há uma baixa cobertura das consultas às gestantes, conforme identificado em estudo anterior^(9)^.

Ao analisar as temáticas das orientações recebidas, observa-se que as prevalências mais baixas estão relacionadas às orientações sobre sinais de trabalho de parto e sinais de risco na gravidez. As orientações específicas da gestação, parto e puerpério podem contribuir para redução no tempo de internação das parturientes e, como consequência, diminuir os riscos de intervenções desnecessárias durante o trabalho de parto e parto, garantindo desfechos obstétricos e perinatais mais favoráveis^(21)^. Ressalta-se a necessidade de abordar orientações específicas sobre a via de parto, como prevenção quaternária e para escolha informada das mulheres, diante de contextos de altas taxas de cesariana^(22)^, como no Brasil.

Quanto à orientação sobre a maternidade de referência, houve um aumento da prevalência do ano de 2013 para 2019, evidenciando um avanço no recebimento dessa orientação. A orientação sobre a maternidade de referência tem o objetivo de facilitar o acesso da usuária ao serviço de saúde no momento do parto ou diante de intercorrências, buscando, assim, reduzir a peregrinação da gestante na rede, que ainda apresenta taxas elevadas no país^(23,24)^.

A vinculação da gestante com a maternidade foi preconizada com a implantação da Rede Cegonha, e deve ocorrer desde a primeira consulta de pré-natal, favorecendo referência e contrarreferência, bem como a alta responsável e a continuidade da assistência após o parto^(7)^. Mais recentemente, a Rede Alyne, instituída pelo Ministério da Saúde, reestrutura a Rede Cegonha com objetivo de consolitar um modelo de cuidado humanizado e integral para gestantes, parturientes, puérperas e crianças^(25)^, na direção da eliminação da peregrinação para o parto e redução das mortes maternas, incluindo o componente de desigualdade racial em destaque.

As orientações relacionadas aos hábitos de vida, como manter uma alimentação saudável, não fumar e não beber, apresentaram prevalência elevada. A adesão a um estilo de vida saudável na gravidez está associada a uma redução no risco de doenças, como o diabetes gestacional e o risco de alterações, no desenvolvimento psicomotor da criança e, portanto, deve ser considerada como uma estratégia eficaz na prevenção de agravos^(26)^.

Observou-se ainda que, no questionário da PNS de 2013, as orientações sobre risco de pressão arterial elevada e risco de açúcar elevado no sangue apresentaram prevalência acima de 90%, sendo duas das orientações mais frequentes neste estudo. No entanto, essas orientações foram ofertadas, principalmente, àquelas mulheres com o diagnóstico dessas comorbidades, evidenciando uma abordagem biologicista na assistência pré-natal, conforme evidenciado em estudos prévios^(7)^. Assim, é necessário que as informações ofertadas às gestantes não sejam apenas para controle de riscos, mas que viabilizem uma educação em saúde como instrumento de construção dialógica do conhecimento, com estímulo à autonomia, participação popular e protagonismo das mulheres no seu próprio cuidado^(27)^.

Em relação às variáveis assistenciais, foi observado que ser atendida no sistema público de saúde está associada a menor prevalência de receber orientações. Tal achado evidencia que essa variável possa ter funcionado mais como um marcador social do que assistencial, tendo em vista que mulheres assistidas nesse serviço apresentam maior vulnerabilidade social^(16)^ e, como apresentado anteriormente, gestantes expostas às condições de desigualdade estão sujeitas a receber com menor frequência as orientações no pré-natal.

Assim, os nossos achados demonstram iniquidades, pois mulheres com alguma vulnerabilidade social estão recebendo menor cuidado ou assistência com frequência menor do que as demais, o que aponta para a necessidade de desenvolver estratégias direcionadas para as populações menos favorecidas, buscando facilitar o contato com os serviços de saúde e início precoce no pré-natal, bem como investir na qualificação dos profissionais de saúde para melhor compreensão do seu papel na oferta de um cuidado equitativo, comprometido com o enfrentamento das desigualdades e com a transformação social. A Rede Alyne prevê essas necessidades, e o presente estudo pode se tornar um ponto de referência para avaliações futuras com a implementação das novas estratégias. Nesse sentido, também se faz necessário destacar a importância do profissional de enfermagem, no cenário internacional e nacional, que tem em seu escopo de atuação as relações interpessoais e educativas em saúde para além do controle da doença, suscitando promoção de estilos de vida saudáveis, socialização de conhecimentos e aormação de sujeitos críticos e autônomos no cuidado de si^(27)^. Os enfermeiros são responsáveis pelo cuidado pré-natal na maioria dos países, incluindo as enfermeiras obstétricas, e, portanto, ampliar sua habilidade para a promoção da saúde e mudanças no comportamento em saúde é fundamental^(27)^.

Portanto, a identificação das condições de desigualdades no acesso aos cuidados de saúde, conforme levantamento desta pesquisa, contribui para informar intervenções e políticas públicas específicas destinadas à constante melhoria da assistência ofertada e, consequentemente, redução da morbimortalidade materna e neonatal^(28)^. Apesar dos avanços na atenção obstétrica no país e da evidência deste estudo da prevalência alta de orientações durante o pré-natal, mudanças políticas como a recente interrupção da Rede Cegonha pela Rede de Atenção Materna e Infantil (Rami), com foco uniprofissional, médico centrado e desarticulado com demais esferas do governo^(29)^, em um contexto político conservador que interrompeu várias políticas para garantia dos direitos das mulheres, juntamente com o desfinanciamento do SUS^(12,30)^ e a pandemia, podem ter contribuido para o aprofundamento dessas desigualdades. Essa realidade pode ser inclusive a vivenciada por outros países. Portanto, estudos futuros serão necessários para o contínuo monitoramento desta assistência no país, principalmente com a instituição da nova estratégia governamental, com vistas ao enfrentamento desses persistentes e agravados retrocessos.

Em relação às limitações do estudo, reconhece-se a possibilidade de viés de memória nos dados levantados, uma vez que, nas entrevistas da PNS, foram questionadas informações sobre o parto nos últimos dois anos. Outra limitação é a não representatividade amostral da população amarela e indígena, cujos dados devem ser interpretados com cautela. Ademais, não puderam ser avaliados avanços ou retrocessos quanto ao recebimento da maioria das orientações abordadas na PNS 2013, com exceção da orientação sobre maternidade de referência, uma vez que as demais orientações não foram incluídas no questionário da PNS 2019. Além de não permitir o monitoramento desses indicadores, a não inclusão das orientações no questionário reforça um cuidado, exclusivamente, centrado nas técnicas e aspectos biológicos. Aponta ainda para desvalorização da educação em saúde como tecnologia leve do cuidado, retratada neste estudo como eixo norteador da prática profissional para promoção e recuperação da saúde, e emancipação da gestante no processo de cuidado.

## CONCLUSÃO

Os resultados deste estudo mostram que as orientações no pré-natal estão sendo realizadas pelos profissionais, com destaque para orientação sobre maternidade de referência que apresentou um aumento significativo da prevalência do ano de 2013 para 2019. Entretanto, apontam para desigualdades e iniquidades dessa assistência, como menor orientação recebida pelas mulheres com baixa escolaridade, pardas e pretas, que residem na região Nordeste, que realizaram o PN no sistema de saúde público e, principalmente, com menor prevalência de orientações quanto às especificidades do ciclo gravídico-puerperal. Com isso, acredita-se que estudos como esse, que avaliam as orientações que estão sendo fornecidas às gestantes durante o acompanhamento pré-natal no âmbito nacional e que abrangem um maior número de orientações, podem contribuir para uma melhor avaliação da realidade dessa assistência. Soma-se a isso a importância de fortalecer a participação do enfermeiro na oferta de um cuidado integral para a saúde da mulher, com enfoque na promoção da saúde e maior comprometimento com o enfrentamento das condições de desigualdades na oferta das orientações durante o pré-natal. O presente estudo pode se tornar um ponto de referência para avaliações futuras, tendo em vista os retrocessos político-programáticos no âmbito da saúde das mulheres no país, que também podem ser a realidade de outros países, tendo em vista o crescente conservadorismo, pandemia, guerras e situações que colocam em maior risco a saúde das mulheres e a garantia dos seus direitos.

# Translation: Orientaciones recibidas en el cuidado prenatal de mujeres brasileñas

**DOI:** 10.1590/1980-220X-REEUSP-2024-0198es

**Authors:** Ana Clara Alves Tomé de Souza, Marina Garcia Avelar Rocha, Kênia Lara Silva, Rafaela Siqueira Costa Schreck, Eunice Francisca Martins, Nágela Cristine Pinheiro Santos, Mariana Santos Felisbino-Mendes

**Affiliations:** 1 Universidade Federal de Minas Gerais, Escola de Enfermagem, Belo Horizonte, MG, Brazil.; 2 Universidade Federal de Minas Gerais, Escola de Enfermagem, Departamento de Enfermagem Aplicada, Belo Horizonte, MG, Brazil.; 3 Universidade Federal de Minas Gerais, Escola de Enfermagem, Departamento de Enfermagem Materno-Infantil e Saúde Pública, Belo Horizonte, MG, Brazil

**Keywords:** Educación en Salud, Atención Prenatal, Factores Socioeconómicos, Salud de la Mujer, Inequidades en Salud

## Abstract

**Objetivo:**

Analizar las orientaciones recibidas durante el prenatal por mujeres brasileñas y su asociación con características sociodemográficas y de atención.

**Método:**

Estudio transversal, que utilizó datos de las ediciones 2013 y 2019 de la Encuesta Nacional de Salud. Se estudiaron mujeres de 18 a 49 años que recibieron atención prenatal dentro de los dos años anteriores a la encuesta (1.851 en 2013 y 2.819 en 2019). Las prevalencias se estimaron con sus Intervalos de Confianza del 95% para las diferentes guías. Luego se estimaron la prevalencia, la Razón de Prevalencia no ajustada y el Intervalo de Confianza del 95% según variables sociodemográficas y asistenciales.

**Resultados:**

Los resultados revelan desigualdades en la orientación recibida durante el control prenatal, principalmente entre mujeres que viven en regiones menos desarrolladas del país, tienen color de piel parda/negra, tienen baja escolaridad y que realizan el control prenatal en el sistema público de salud.

**Conclusión:**

Las orientaciones son brindadas por profesionales, con énfasis en la orientación sobre la maternidad de referencia, pero existen inequidades en la provisión de estas orientaciones.

## INTRODUCCIÓN

La asistencia obstétrica debe basarse en la atención interprofesional prestada a las mujeres durante la atención prenatal, el parto y el puerperio. Es necesario integrar a las mujeres como protagonistas de estos cuidados y de sus elecciones, con acceso a acciones de cribado, diagnóstico y prevención de enfermedades, además de información sobre aspectos fisiológicos y comportamentales, basada en evidencias científicas, así como apoyo en relación con cuestiones sociales y emocionales^(1)^. Así, el uso de tecnologías blandas, como la escucha cualificada y los cuidados humanizados^(2)^, inherentes a las buenas prácticas obstétricas, puede ayudar a las mujeres a vivir la maternidad de forma positiva, lo que implica autoestima, competencia y autonomía, resultantes de la seguridad, el respeto y la dignidad, a través de la comunicación, la acogida y la educación para la salud^(1)^. En este sentido, la educación para la salud se considera una práctica social y el principio rector de la atención prenatal^(3)^. Desde esta perspectiva, se entiende que todas las consultas son oportunidades para realizar intervenciones educativas individuales o colectivas, que también han sido recomendadas para reparar vulnerabilidades durante el embarazo^(4,5)^. Para ello, la educación en salud asume un compromiso humanista y transformador que, bajo una concepción freireana, debe sustentarse en el diálogo entre educador y educando, en un proceso multidimensional que considere aspectos culturales, económicos y sociales^(6)^. Sin embargo, estudios han identificado que los profesionales de salud no han realizado o han realizado parcialmente la educación para la salud en la atención a gestantes^(7,8)^. Al evaluar la calidad de la atención prenatal en la red de atención primaria de Brasil, se observó que sólo el 60% de las embarazadas recibían orientación sobre cuidados prenatales, como lactancia materna exclusiva, nutrición y aumento de peso, cuidados del recién nacido e importancia de la citología vaginal^(9)^. Otros hallazgos también han identificado un acceso limitado a la orientación sobre el parto^(10)^ y sobre el hospital de maternidad de referencia^(11)^, lo que apunta a un déficit en la educación sanitaria durante la atención prenatal. Cuando se identificó la completitud de las acciones y orientaciones recomendadas durante el control prenatal, fue significativamente mayor para las gestantes de mayor edad y renta, y en los municipios más grandes^(9)^. Este hallazgo revela que persisten desigualdades sociales e individuales en la calidad de la atención prenatal ofrecida a las mujeres en el país, poniendo en riesgo la garantía del principio de equidad en el Sistema Único de Salud (SUS). Además, persisten modelos de atención centrados en la enfermedad y en procedimientos técnicos, en detrimento de un modelo de atención basado en la atención integral de la salud con acciones de educación sanitaria^(12)^. De esta forma, la orientación prestada a las gestantes es un indicador que puede evaluar la calidad de la atención prenatal, dada su asociación directa con la promoción de la salud. Sin embargo, la mayoría de los estudios encontrados en la literatura evalúan la calidad de la atención prenatal de forma local/regional y solamente por medio de marcadores de acceso y/o marcadores clínicos^(11,13–16)^. Cuando se evalúan, las directrices se abarcan en menor número, y a menudo no se identifican los subgrupos más afectados por la falta de atención, hecho que también puede observarse en los estudios nacionales^(12,15)^.

En vista de lo anterior, este estudio se centró en las orientaciones dadas a las embarazadas durante la atención prenatal. Estudios de esta naturaleza pueden contribuir a una mejor evaluación de la realidad de la asistencia prenatal a nivel poblacional, evaluando elementos distintos de los marcadores de acceso. Por lo tanto, el objetivo de este estudio fue analizar la orientación recibida durante el control prenatal por las mujeres brasileñas y su asociación con las características sociodemográficas y asistenciales.

## MÉTODO

### Tipo de Estudio

Se trata de un estudio transversal con datos de la Encuesta Nacional de Salud (ENS), ediciones 2013 y 2019, que es una encuesta brasileña cuyo objetivo principal es caracterizar la situación de salud y los estilos de vida de la población brasileña.

### Definición de la Muestra

El proceso de muestreo de la ENS se realizó en tres etapas: en la primera, se seleccionaron los distritos censales; en la segunda, se seleccionaron los hogares; y en la tercera, por cada hogar, se seleccionó un residente de 18 años o más en 2013, y de 15 años o más en 2019, todo ello por muestreo aleatorio simple. El cuestionario ENS en ambas ediciones se dividió en módulos que cubrían las características del hogar, de todos los residentes y del residente adulto seleccionado. En el módulo S, se investigó la atención prenatal entre las mujeres que dieron a luz entre el 28/07/2011 y el 27/07/2013 en la edición 2013, y en el periodo igual o posterior al 28/07/2017 en la edición 2019, es decir, en los últimos dos años antes de las encuestas, considerando sólo el último parto para responder las preguntas. Entre las mujeres que respondieron el módulo S de los cuestionarios, se definió una submuestra que incluyó sólo a las mujeres de 18 a 49 años que tuvieron control prenatal durante su último embarazo, totalizando 1.851 mujeres disponibles para el estudio en 2013 y 2.819 mujeres en 2019.

### Recogida de Datos

En este estudio se analizaron diez preguntas específicas relativas a la orientación proporcionada durante la atención prenatal, a saber: durante sus citas de atención prenatal, ¿recibió alguno de los siguientes consejos: no faltar a las citas, llevar una dieta saludable, no fumar, no beber, sobre lactancia materna, signos de riesgo en el embarazo, signos de parto, riesgos de hipertensión arterial, riesgos de hiperglucemia y a qué servicio sanitario debe acudir en el momento del parto?

Sin embargo, se observó que, en comparación con 2013, solo se mantuvo en 2019 una de las directrices analizadas anteriormente, que corresponde a la orientación sobre a qué servicio sanitario de referencia debe acudir la mujer en el momento del parto. Las demás orientaciones no fueron investigadas, poniendo en riesgo el seguimiento de estos indicadores. La orientación fue considerada la variable dependiente y las siguientes variables sociodemográficas fueron utilizadas como variables independientes: grupo de edad (18–29 años, 30–39 años y 40–49 años); región (Norte, Nordeste, Centro-Oeste, Sudeste y Sur); escolaridad, en años (0–8 años, 9–11 años o 12 o más); color de piel (blanca, amarilla, parda, negra e indígena); y lugar de atención prenatal (público, privado u otro sistema de salud).

### Análisis Y Recopilación de Datos

En primer lugar, se estimaron las tasas de prevalencia con sus Intervalos de Confianza al 95% para las diferentes orientaciones recibidas por las mujeres en 2013 y 2019. A continuación, se estimaron estas prevalencias en función de variables sociodemográficas y asistenciales. Para evaluar las diferencias estadísticas entre las prevalencias se utilizó la prueba de chi-cuadrado de Pearson. Finalmente, se estimó la Razón de Prevalencia No Ajustada (RPna) y el Intervalo de Confianza del 95% de haber recibido asesoramiento para los diferentes grupos de variables independientes sólo para los factores que mostraron un valor p < 0,2 en el análisis descriptivo. Los datos se analizaron con el programa estadístico Stata, versión 14.2, utilizando el módulo de *encuestas* para obtener estimaciones poblacionales, considerando estrato, conglomerado y peso de los individuos de la muestra compleja.

### Aspectos Éticos

Este estudio utiliza datos secundarios de la ENS, que están disponibles públicamente en los sitios web oficiales del Ministerio de Salud de Brasil o del Instituto Brasileño de Geografía y Estadística, y están exentos de ser analizados por un Comité de Ética en Investigación, de acuerdo con la Resolución 510/2016 del Consejo Nacional de Salud. La ENS fue aprobada por el Comité Nacional de Ética en Investigación en julio de 2013 y en agosto de 2019.

## RESULTADOS

Se observa que tanto en 2013 como en 2019, respectivamente, la mayoría de las mujeres que habían dado a luz en los últimos dos años previos a las encuestas tenían entre 18 y 29 años (61,2%/52,7%), vivían en el Sudeste (38,1%/36, 9%), seguido del Nordeste (29,1%/29,5%), tenían entre 9 y 11 años de escolaridad (47,2%/8,6%), se autodeclaraban pardas (50,0%/51,7%) y utilizaban el servicio público de salud para la atención prenatal (71,9%/70%) (Tabla 1).

**Tabla 1:** Prevalencia e intervalos de confianza del 95% de las características sociodemográficas y de atención sanitaria de las mujeres brasileñas que tuvieron atención prenatal en Encuesta Nacional de Salud – Brasil, 2013 y 2019.

Variables Sociodemográficas y asistenciales	2013		2019	
	n*	%** (IC95%)	n*	%** (IC95%)
**Grupos de edad (años)**				
18–29	1,120	61,2 (57,4–64,9)	1,456	52,7 (49,3–55,9)
30–39	662	35,2 (31,7–38,9)	1,124	41,5 (38,3–44,8)
40–49	69	3,6 (2,5–5,1)	149	5,7 (4,4–7,3)
**Región**				
Centro–Oeste	232	8,2 (7,0–9,6)	344	9,2 (7,9–10,8)
Norte	517	9,9 (8,5–11,5)	687	11,6 (10,3–13,1)
Noreste	565	29,1 (25,9–32,7)	1,041	29,5 (26,9–32,2)
Sudeste	345	38,1 (34,1–42,3)	476	36,9 (33,4–40,5)
Sur	192	14,6 (12,1–17,5)	271	12,5 (10,8–14,5)
**Escolaridad (años)**				
0–8	707	36,7 (33,1–40,0)	900	27,6 (24,8–30,6)
9–11	830	47,2 (43,4–51,1)	1,310	48,6 (45,3–51,8)
12 o +	314	16,1 (13,5–19,1)	609	23,7 (21,0–26,6)
**Color/raza**				
Blanca	593	39,9 (36,1–43,8)	802	35,1 (32,0–38,3)
Negra	167	8,8 (6,9–11,2)	293	11,8 (9,6–14,3)
Parda	1,052	50,0 (46,2–53,8)	1,665	51,7 (48,4–54,9)
Amarilla	24	1,1 (0,006–2,1)	31	1,0 (0,6–1,5)
Indígena	15	0,1 (0,0006–0,6)	28	0,6 (0,3–1,0)
**Local de realización del prenatal**				
**Público**	1,401	71,9 (68,0–75,5)	2,141	70,0 (66,7–73,1)
**Privado**	435	27,3 (23,8–31,2)	677	29,9 (26,8–33,2)
**Otros**	15	0,7 (0,3–1,5)	1	0,1 (0–1,0)

Nota: *n de la muestra; ** prevalencia poblacional; IC95%: Intervalo de 95% Confianza

Foi observado um aumento significativo da prevalência da orientação sobre a maternidade de referência de 2013 (74,9%) para 2019 (83,0%) (Tabla 2). Entretanto, observaram-se desigualdades na oferta dessa orientação nos dois períodos, uma vez que as mulheres da faixa etária de 18–29 anos, que viviam nas regiões Norte e Nordeste, com baixa escolaridade, indígenas, pretas e pardas, que realizaram o pré-natal no sistema de saúde público, apresentaram menor prevalência de serem orientadas.

**Tabla 2:** Prevalência, Razão de Prevalência não ajustada e Intervalos de Confiança de 95% das orientações gerais recebidas durante o cuidado pré-natal das mulheres brasileiras com parto nos últimos dois anos antes dos inquéritos en Encuesta Nacional de Salud – Brasil, 2013 y 2019.

Variables Sociodemográficas y asistenciales	Maternidad de referencia/2013			Maternidad de referencia/2019			No faltar a las consultas		
	n*	%** (IC95%)	RPna (IC95%)	n*	%**(IC95%)	RPna (IC95%)	n*	%**(IC95%)	RPna (IC95%)
**Recibió orientación**	1,362	74,9 (71,4–78,1)	-	2,244	83,0 (80,7–85,1)	-	1,680	88,9 (85,8–91,4)	-
**Grupos de edad (años)**		p*** = 0,011			p*** = 0,0006			p*** = 0,338	
18–29	807	71,1 (66,3–75,5)	Ref.	1,113	79,0 (75,3–82,3)	Ref.	1,018	87,5 (83,1–90,9)	-
30–39	505	81,1 (76,0–85,4)	**1,14 (1,04–1,24)**	939	88,2 (84,9–90,8)	**1,08 (1,02–1,15)**	598	91,5 (87,4–94,3)	-
40–49	50	77,0 (58,3–88,9)	1,08 (0,87–1,33)	124	85,5 (75,0–92,1)	1,15 (0,99–1,34)	64	87,0 (63,7–96,3)	-
**Región**		p*** < 0,0001			p***< 0,0001			p***= 0,530	
Centro–Oeste	168	71,2 (62,9–78,2)	Ref.	298	87,9 (82,3–91,8)	Ref.	203	86,0 (79,5–90,7)	-
Norte	380	72,6 (64,9–79,1)	1,02 (0,88–1,18)	504	72,4 (66,6–77,6)	0,88 (0,80–0,98)	478	93,4 (89,1–96,1)	-
Noreste	368	61,0 (53,9–67,7)	0,85 (0,73–1,00)	781	75,9 (71,6–79,7)	0,87 (0,81–0,94)	516	90,0 (83,6–94,3)	-
Sudeste	279	83,6 (77,5–88,3)	**1,17 (1,03–1,33)**	410	87,7 (82,6–91,5)	1,01 (0,93–1,08)	309	87,3 (81,0–91,7)	-
Sur	167	83,3 (74,2–89,7)	**1,17 (1,01–1,34)**	251	92,3 (87,6–95,4)	1,09 (1,00–1,18)	174	89,0 (79,8–94,3)	-
**Escolaridad (años)**		p*** = 0,051			p*** = 0,0002			p*** = 0,656	
0–8	491	69,4 (63,7–74,6)	Ref.	675	78,0 (73,6–81,9)	Ref.	652	90,4 (85,1–93,9)	-
9–11	620	77,7 (72,6–82,0)	**1,11 (1,01–1,23)**	1,026	81,7 (78,0–84,9)	1,02 (0,95–1,10)	754	87,7 (82,6–91,5)	-
12 o +	251	79,0 (69,6–86,0)	1,13 (1,00–1,29)	543	91,5 (87,0–94,5)	**1,11 (1,03–1,19)**	274	88,8 (82,0–93,2)	-
**Color/raza**		p*** < 0,0001			p*** = 0,008			p*** = 0,735	
Blanca	475	84,0 (79,2–87,8)	Ref.	683	88,7 (84,8–91,7)	Ref.	534	89,8 (85,0–93,2)	-
Preta	116	72,6 (61,9–81,2)	1,09 (1,00–1,19)	238	84,3 (75,1–90,6)	1,04 (0,97–1,12)	156	87,6 (72,9–94,9)	-
Parda	743	68,5 (63,3–73,3)	**1,13 (1,07 –1,19)**	1,270	80,4 (77,2–83,3)	**1,07 (1,03–1,12)**	952	88,3 (83,9–91,6)	-
Amarilla	17	79,2 (52,9–92,8)	1,04 (0,88–1,23)	25	86,0 (64,1–95,4)	1,02 (0,90–1,17)	24	100,0	-
Indígena	11	54,3 (18,0–86,6)	1,26 (0,94–1,68)	25	98,0 (91,9–99,5)	**0,92 (0,88–0,96)**	14	97,3 (78,9–99,7)	-
**Local de realización del prenatal**	p*** < 0,0001			p*** = 0,002			p*** = 0,137	
Público	993	69,4 (65,0–73,5)	Ref.	1,619	79,9 (77,1–82,4)	Ref.	1,286	89,5 (85,9–92,3)	-
Privado	356	88,7 (84,0–92,0)	**1,27 (1,18–1,38)**	614	90,3 (85,7–93,6)	**1,07 (1,01–1,13)**	384	88,0 (81,2–92,5)	-
Otros	13	95,8 (80,7–99,2)	**1,38 (1,25–1,51)**	01	100,0	**1,18 (1,14–1,22)**	10	60,9 (23,5–88,8)	-

Nota: *n de la muestra; ** prevalencia poblacional; *** test chi cuadrado de Pearson; IC95%: Intervalo de Confianza de 95%; RPna: Razón de Prevalencia no ajustada.

Hubo un aumento significativo en la prevalencia de orientación maternal de referencia de 2013 (74,9%) a 2019 (83,0%) (Tabla 2). Sin embargo, hubo desigualdades en la prestación de esta orientación en los dos períodos, pues las mujeres del grupo de 18 a 29 años, residentes en las regiones Norte y Nordeste, con baja escolaridad, indígenas, negras y pardas, que tuvieron atención prenatal en el sistema público de salud, presentaron menor prevalencia de ser orientadas.

La orientación de no faltar a las citas tuvo una alta prevalencia (88,9%). No hubo asociación entre las variables sociodemográficas y sanitarias y este consejo. En cuanto a la orientación sobre hábitos de vida, como mantener una dieta saludable, no fumar y no beber, independientemente de las variables sociodemográficas y sanitarias, la prevalencia de haber recibido esta orientación fue superior al 80%, siendo la orientación sobre alimentación saludable la más frecuente de ellas (96,8%) (Tabla 3).

**Tabla 3:** Prevalencia, razón de prevalencia no ajustada e intervalos de confianza del 95% de la orientación recibida sobre hábitos de vida durante la atención prenatal entre las mujeres brasileñas que dieron a luz en los últimos dos años antes de las encuestas en Encuesta Nacional de Salud – Brasil, 2013 y 2019.

Variables Sociodemográficas y asistenciales	Alimentación saludable			No fumar			No beber		
	n*	%** (IC95%)	RPna (IC95%)	n*	%**(IC95%)	RPna (IC95%)	n*	%**(IC95%)	RPna (IC95%)
**Recibió orientación**	1,792	96,8 (95,2–97,9)	-	1,684	89,1 (86,0–91,6)	-	1,687	89,9 (87,1–92,2)	-
**Grupos de edad (años)**		p*** = 0,491			p*** = 0,824			p*** = 0,291	
18–29	1,080	96,4 (94,0–97,8)	-	1,015	88,6 (84,7–91,6)	-	1,019	89,2 (85,4–92,2)	Ref.
30–39	643	97,2 (94,8–98,5)	-	603	89,7 (83,7–93,7)	-	602	90,3 (85,3–93,7)	1,02 (0,96–1,09)
40–49	69	100	-	66	92,8 (66,9–98,8)	-	66	97,5 (91,1–99,3)	1,00 (0,91–1,10)
**Región**		p*** = 0,361			p*** = 0,153			p*** = 0,559	
Centro–Oeste	226	96,4 (91,9–98,4)	-	211	88,6 (80,1–93,7)	Ref.	210	88,6 (80,2–93,7)	-
Norte	501	98,0 (96,3–99,0)	-	482	92,9 (86,5–96,3)	1,04 (0,96–1,14)	482	93,0 (86,9–96,4)	-
Noreste	548	98,0 (96,2–99,0)	-	516	92,4 (86,5–95,8)	1,04 (0,95–1,14)	513	91,5 (85,7–95,1)	-
Sudeste	335	96,4 (92,6–98,3)	-	299	85,4 (79,0–90,1)	0,96 (0,87–1,06)	302	86,7 (82,1–91,7)	-
Sur	182	94,8 (87,6–97,9)	-	176	89,9 (81,5–94,8)	1,01 (0,91–1,12)	180	91,0 (81,8–95,8)	-
**Escolaridad (años)**		p*** = 0,072			p*** = 0,970			p*** = 0,706	
0–8	685	97,1 (94,8–98,4)	Ref.	646	88,8 (93,5–92,5)	-	648	88,7 (83,4–92,5)	-
9–11	798	95,8 (92,6–97,6)	0,98 (0,95–1,01)	754	89,5 (84,8–92,8)	-	755	91,0 (87,3–93,7)	-
12 o +	309	99,2 (97,5–99,8)	1,02 (1,00–1,04)	284	88,9 (79,8–94,2)	-	284	89,3 (80,5–94,4)	-
**Color/raza**		p*** = 0,890			p*** = 0,494			p*** = 0,258	
Blanca	574	96,3 (92,7–98,2)	-	535	90,1 (85,9–93,1)	-	541	91,9 (88,0–94,6)	-
Preta	162	97,1 (92,1–99,0)	-	151	90,9 (79,7–96,2)	-	149	90,3 (79,4–95,8)	-
Parda	1,018	97,0 (94,8–98,2)	-	960	87,3 (82,0–91,1)	-	959	87,6 (83,0–91,1)	-
Amarilla	24	100,0	-	24	100,0	-	24	100,0	-
Indígena	14	97,3 (78,9–99,7)	-	14	97,3 (78,9–99,7)	-	14	97,3 (78,9–99,7)	-
**Local de realización del prenatal**	p*** = 0,105			p*** = 0,542			p*** = 0,116	
Público	1,352	96,2 (94,0–97,6)	Ref.	1,278	89,0 (85,3–91,8)	-	1,277	88,8 (85,1–91,7)	Ref.
Privado	426	98,2 (95,8–99,3)	1,02 (0,99–1,04)	392	89,2 (82,4–93,6)	-	396	92,5 (88,0–95,3)	1,04 (0,98–1,09)
Otros	18	99,4 (95,3–99,9)	1,03 (1,01–1,05)	14	99,4 (95,3–99,9)	-	14	99,4 (95,3–99,9)	1,11 (1,07–1,16)

Nota: *n de la muestra; ** prevalencia poblacional; *** test chi cuadrado de Pearson; IC95%: Intervalo de Confianza de 95%; RPna: Razón de Prevalencia no ajustada.

También se observa que la prevalencia de orientación sobre el riesgo de hipertensión arterial fue del 91,2% y sobre el riesgo de hiperglucemia del 92,9%, lo que demuestra que fueron frecuentes para la mayoría de las mujeres que presentaron hipertensión arterial y alteraciones de la glucemia durante el embarazo, ya que sólo se preguntó por esta orientación a las que presentaron estas alteraciones (Tabla 4). No hubo asociación entre las variables sociodemográficas y asistenciales y recibir consejo sobre hábitos de vida y riesgos durante el embarazo, a excepción del consejo sobre el riesgo de hiperglucemia, en el que la atención prenatal prestada por el sistema público de salud se asoció con una menor prevalencia de recibir este consejo (Tablas 3 y 4).

**Tabla 4:** Prevalencia, razón de prevalencia no ajustada e intervalos de confianza del 95% de la orientación recibida sobre los riesgos durante el embarazo a través de la presión arterial elevada y la glucemia entre las mujeres brasileñas que dieron a luz en los últimos dos años antes de las encuestas en Encuesta Nacional de Salud – Brasil, 2013 y 2019.

Variables Sociodemográficas y asistenciales	Riesgo de hipertensión			Riesgo de hiperglucemia		
	n*	%** (IC95%)	RPna (IC95%)	n*	%**(IC95%)	RPna (IC95%)
**Recibió orientación**	347	91,2 (85,7–94,7)	-	89	92,9 (86,0–96,5)	-
**Grupos de edad (años)**		p*** = 0,719			p*** = 0,671	
18–29	191	90,0 (82,0–94,7)	-	42	90,6 (78,5–96,2)	-
30–39	134	92,6 (83,0–96,9)	-	40	94,1 (80,9–98,4)	-
40–49	22	92,7 (69,8–98,6)	-	7	100,0	-
**Región**		p*** = 0,610			p*** = 0,177	
Centro–Oeste	50	94,1 (82,1–98,3)	-	8	77,0 (38,0–94,8)	Ref.
Norte	102	89,9 (81,7–94,7)	-	27	87,7 (69,9–95,7)	1,14 (0,75–1,72)
Noreste	106	94,2 (88,7–97,1)	-	18	92,1 (68,2–98,5)	1,19 (0,79–1,80)
Sudeste	51	90,6 (78,3–96,3)	-	19	95,7 (82,2–99,1)	1,24 (0,83–1,84)
Sur	38	85,7 (58,3–96,5)	-	17	98,9 (91,7–99,8)	1,28 (0,86–7,89)
**Escolaridad (años)**		p*** = 0,921			p*** = 0,553	
0–8	145	90,0 (79,7–95,4)	-	29	91,8 (76,7–97,4)	-
9–11	150	92,0 (83,6–96,3)	-	36	91,3 (79,0–96,7)	-
12 o +	52	91,1 (70,5–97,8)	-	24	100,0	-
**Color/raza**		p*** = 0,473			p*** = 0,936	
Blanca	103	87,6 (74,7–94–4)	-	27	92,5 (73,1–98,2)	-
Preta	31	93,6 (80,8–98,1)	-	4	88,8 (45,0–98,7)	-
Parda	203	92,9 (85,5–96,6)	-	54	92,7 (83,7–96,9)	-
Amarilla	4	100,0	-	3	100,0	-
Indígena	6	70,7 (14,9–97,1)	-	1	100,0	-
**Local de realización del prenatal**		p*** = 0,092			p***<0,0001	
Público	275	89,4 (82,5–93,7)	Ref.	61	91,0 (81,4–95,9)	Ref.
Privado	71	96,6 (82,9–99,4)	1,08 (0,99–1,17)	28	99,0 (92,6–99,9)	1,17 (1,08–1,28)
Otros	01	41,1 (4,2–91,8)	0,46 (0,08–2,35)	0	0	-

Nota: *n de la muestra; ** prevalencia poblacional; *** test chi cuadrado de Pearson; IC95%: Intervalo de Confianza de 95%; RPna: Razón de Prevalencia no ajustada.

En relación a todas las orientaciones estudiadas, hubo menor prevalencia de orientaciones específicas al ciclo embarazo-puerperio, como señales de trabajo de parto (70,8%) y señales de riesgo en el embarazo (75,2%), siendo la orientación sobre señales de trabajo de parto la menos prevalente (Tablas 2 y 5). También se observó que algunos subgrupos poblacionales recibieron menos orientaciones específicas para el embarazo que otros, como las mujeres negras, pardas, amarillas e indígenas, con 0 a 8 años de escolaridad, que vivían en el Nordeste y tenían atención prenatal en el sistema público de salud (Tabla 5), tal como se observó con relación a la maternidad de referencia. Llama la atención que cuando estas orientaciones se relacionan con la escolaridad, la prevalencia es 15% menor entre las mujeres con baja escolaridad en comparación con las de alta escolaridad (Tabla 5).

**Tabla 5:** Prevalencia, razón de prevalencia no ajustada e intervalos de confianza del 95% de la orientación específica sobre el ciclo embarazo-puerperio recibida durante la atención prenatal entre las mujeres brasileñas que dieron a luz hasta dos años antes de la encuesta en Encuesta Nacional de Salud – Brasil, 2013 y 2019.

Variables Sociodemográficas y asistenciales	Signos de trabajo de parto			Signos de riesgo en el embarazo			Lactancia materna		
	n*	%** (IC95%)	RPna (IC95%)	n*	%**(IC95%)	RPna (IC95%)	n*	%**(IC95%)	RPna (IC95%)
**Recibió orientación**	1,320	70,8 (67,3–74,2)	-	1,403	75,2 (71,7–78,4)	-	1,543	82,4 (79,7–85,2)	-
**Grupos de edad (años)**		p*** = 0,048			p*** = 0,019			p*** = 0,123	
18–29	796	68,7 (63,9–73,2)	Ref.	831	72,0 (67,1–76,3)	Ref.	932	80,0 (75,5–83,8)	Ref.
30–39	476	75,9 (70,4–80,6)	1,10 (1,00–1,22)	517	79,9 (74,6–84,4)	1,11 (1,01–1,21)	553	86,4 (81,6–90,2)	1,08 (1,00–1,16)
40–49	48	57,8 (38,8–74,7)	0,84 (0,60–1,17)	55	84,2 (70,8–92,2)	1,17 (1,01–1,34)	58	82,4 (79,0–85,2)	1,03 (0,85–1,25)
**Región**		p*** = 0,028			p*** = 0,088			p*** = 0,288	
Centro–Oeste	155	67,4 (59,9–74,3)	Ref.	175	74,1 (65,9–80,9)	Ref.	184	80,2 (73,0–85,9)	-
Norte	360	68,4 (60,6–75,4)	1,01 (0,87–1,18)	392	75,5 (67,8–81,9)	1,01 (0,88–1,17)	422	78,7 (69,6–85,6)	-
Noreste	393	64,2 (57,5–70,5)	0,95 (0,82–1,10)	411	68,8 (62,0–74,8)	0,92 (0,90–1,06)	469	78,8 (72,4–84,1)	-
Sudeste	261	74,7 (67,9–80,4)	1,10 (0,96–1,26)	270	78,5 (71,9–83,9)	1,05 (0,93–1,20)	295	85,1 (78,9–89,7)	-
Sur	151	77,5 (69,1–84,2)	1,14 (0,99–1,32)	155	79,6 (70,3–86,6)	1,07 (0,92–1,24)	173	85,9 (76,5–92,0)	-
**Escolaridad (años)**		p*** < 0,0001			p*** = 0,029			p*** = 0,0002	
0–8	467	61,6 (55,7–67,1)	Ref.	514	69,9 (64,0–75,1)	Ref.	559	75,7 (69,8–80,7)	Ref.
9–11	616	74,2 (68,7–79,0)	**1,20 (1,07–1,35)**	635	77,0 (71,9–81,5)	1,10 (0,99–1,21)	710	84,2 (79,2–88,2)	**1,11 (1,01–1,21)**
12 o +	237	82,0 (75,7–87,0)	**1,33 (1,18–1,49)**	254	81,9 (73,6–78,4)	1,17 (1,04–1,31)	274	92,1 (87,5–95,1)	**1,21 (1,12–1,32)**
**Color/raza**		p*** = 0,0001			p*** = 0,015			p*** = 0,012	
Blanca	444	79,3 (74,2–83,6)	Ref.	467	80,9 (75,6–85,3)	Ref.	514	88,1 (83,3–91,7)	Ref.
Preta	115	60,8 (48,4–71,9)	**1,15 (1,05–1,27)**	123	65,4 (52,6–76,3)	**1,13 (1,02–1,25)**	140	75,2 (61,7–85,1)	1,12 (1,00–1,23)
Parda	736	67,0 (61,9–71,6)	**1,10 (1,04–1,16)**	782	72,5 (67,4–77,1)	**1,07 (1,01–1,13)**	857	79,3 (74,8–83,2)	**1,08 (1,03–1,13)**
Amarilla	17	42,5 (17,9–71,6)	**1,30 (1,07–1,58)**	19	58,8 (28,2–77,1)	1,19 (0,95–1,49)	20	77,2 (48,7–92,4)	1,10 (0,91–1,32)
Indígena	8	34,8 (12,6–66,5)	**1,37 (1,14–1,65)**	12	74,7 (44,7–91,5)	1,05 (0,86–1,28)	12	82,6 (33,4–97,8)	1,05 (0,79–1,39)
**Local de realización del prenatal**	p*** = 0,002			p*** = 0,0003			p*** = 0,013	
Público	974	67,1 (62,9–71,1)	Ref.	1,038	71,5 (67,2–75,5)	Ref.	1,155	79,8 (75,9–83,3)	Ref.
Privado	337	80,8 (74,3–85,9)	**1,20 (1,09–1,32)**	354	84,4 (78,4–89,0)	**1,17 (1,08–1,28)**	374	88,6 (82,2–92,9)	**1,10 (1,02–1,19)**
Otros	9	63,6 (24,7–90,3)	0,94 (0,51–1,74)	11	91,2 (71,1–97,7)	**1,27 (1,10–1,46)**	14	96,9 (79,5–99,6)	**1,21 (1,12–1,31)**

Nota: *n de la muestra; ** prevalencia poblacional; *** test chi cuadrado de Pearson; IC95%: Intervalo de Confianza de 95%; RPna: Razón de Prevalencia no ajustada.

La prevalencia de haber recibido orientaciones sobre lactancia materna durante el embarazo fue de 82,4%, pero las mujeres con bajo nivel de escolaridad, de raza negra o parda y que tuvieron atención prenatal en el sistema público de salud recibieron menos de 80% de esas orientaciones.

## DISCUSIÓN

Los resultados revelan desigualdades en la recepción de orientación, especialmente entre las mujeres brasileñas que viven en las regiones menos desarrolladas económicamente del país, con color de piel parda y negra, bajo nivel de escolaridad y que asisten al control prenatal en el sistema público de salud, a pesar de la alta prevalencia de orientación recibida durante el control prenatal. Cabe destacar que las desigualdades pueden constituir vulnerabilidades, en el sentido de mayor susceptibilidad a potenciales daños durante el embarazo y el puerperio, especialmente para las mujeres adolescentes, primíparas, con deficiencias nutricionales, con bajo nivel de escolaridad, migrantes y refugiadas^(5)^.

El hallazgo de este estudio de una menor prevalencia de orientación recibida por estas mujeres corrobora los datos presentados por un estudio transversal de base poblacional que concluyó que, en Brasil, ser negro y ocupar posiciones sociales desfavorables conllevan desventajas en el acceso a la atención prenatal que cumple los criterios establecidos por el Ministerio de Salud^(17)^. Además, las puérperas negras tienen mayor riesgo de tener cuidados prenatales inadecuados, falta de apego a la maternidad, ausencia de acompañante, peregrinaje al parto y menos anestesia local para la episiotomía, lo que revela un racismo internalizado en los profesionales de salud que consideran que las mujeres negras son más resistentes al dolor^(18)^.

Se reconoce que las mujeres negras brasileñas enfrentan condiciones de vulnerabilidad superpuestas, ya que experimentan exclusión social debido al racismo estructural y a las desigualdades de género, lo que requiere acciones estratégicas con un enfoque interseccional para abordar estas condiciones y garantizar la igualdad de calidad en la atención prenatal^(19,20)^. Las desigualdades en la oferta de orientación a las mujeres también están asociadas a la región, lo que puede estar vinculado a la cobertura del control prenatal, ya que en el Norte y Nordeste del país hay una baja cobertura de consultas para gestantes, como se identificó en un estudio anterior^(9)^.

Al analizar los temas de la orientación recibida, se observó que las tasas de prevalencia más bajas estaban relacionadas con la orientación sobre los signos del parto y los signos de riesgo en el embarazo. Las orientaciones específicas para el embarazo, el parto y el puerperio pueden ayudar a reducir la duración de la estancia de las parturientas y, en consecuencia, reducir el riesgo de intervenciones innecesarias durante el parto y el alumbramiento, garantizando unos resultados obstétricos y perinatales más favorables^(21)^. Es importante destacar la necesidad de orientaciones específicas sobre el modo de parto, como prevención cuaternaria y para que las mujeres hagan una elección informada, en contextos con altas tasas de cesárea^(22)^, como en Brasil. En cuanto a la orientación sobre el hospital de maternidad de referencia, hubo un aumento de la prevalencia de 2013 a 2019, lo que muestra un progreso en la recepción de esta orientación. La orientación sobre el hospital de maternidad de referencia tiene como objetivo facilitar el acceso de la usuaria al servicio de salud en el momento del parto o en caso de complicaciones, buscando así reducir el peregrinaje de las gestantes por la red, que aún presenta altas tasas en el país^(23,24)^. La vinculación de las gestantes a la maternidad fue preconizada con la implantación de la Red Cigüeña, y debe tener lugar desde la primera consulta prenatal, favoreciendo la referencia y la contrarreferencia, así como el alta responsable y la continuidad asistencial tras el parto^(7)^. Más recientemente, la Red Alyne, creada por el Ministerio de Salud, reestructura la Red Cigüeña con el objetivo de consolidar un modelo de atención humanizada e integral a las gestantes, parturientas, puérperas y niños^(25)^. Las pautas relacionadas con los hábitos de vida, como seguir una dieta sana, no fumar y no beber, tuvieron una elevada prevalencia. La adherencia a un estilo de vida saludable durante el embarazo se asocia a una reducción del riesgo de enfermedades como la diabetes gestacional y del riesgo de alteraciones en el desarrollo psicomotor del niño, por lo que debe considerarse una estrategia eficaz para prevenir problemas de salud^(26)^.

También se observó que en el cuestionario del PNS de 2013, las orientaciones sobre el riesgo de hipertensión arterial y el riesgo de hiperglucemia tenían una prevalencia superior al 90%, y fueron dos de las orientaciones más frecuentes en este estudio. Sin embargo, estas orientaciones se ofrecieron principalmente a las mujeres diagnosticadas con estas comorbilidades, lo que muestra un enfoque biologicista de la atención prenatal, como se ha puesto de manifiesto en estudios anteriores^(7)^. Por lo tanto, es necesario que la información ofrecida a las gestantes no sea sólo para el control de riesgos, sino que posibilite la educación para la salud como instrumento de construcción dialógica del conocimiento, fomentando la autonomía, la participación popular y el protagonismo de las mujeres en su propio cuidado^(27)^.

Con relación a las variables de cuidado, se observó que ser cuidada en el sistema público de salud está asociado a una menor prevalencia de recibir orientación. Este hallazgo muestra que esta variable puede haber funcionado más como un marcador social que como un marcador de cuidado, dado que las mujeres que reciben atención en este servicio son socialmente más vulnerables^(16)^ y, como se mencionó anteriormente, las gestantes expuestas a condiciones de desigualdad tienen menor probabilidad de recibir orientación para el cuidado prenatal.

Así, nuestros hallazgos demuestran desigualdades, ya que las mujeres con alguna vulnerabilidad social están recibiendo menos atención o con menor frecuencia que otras, lo que apunta a la necesidad de desarrollar estrategias dirigidas a las poblaciones menos favorecidas, buscando facilitar el contacto con los servicios de salud y la atención prenatal precoz, así como invertir en la capacitación de los profesionales de la salud para que comprendan mejor su papel en la prestación de una atención equitativa, comprometida con el abordaje de las desigualdades y la transformación social. La Red Alyne contempla estas necesidades, y este estudio podría convertirse en un punto de referencia para futuras evaluaciones con la implementación de nuevas estrategias. En este sentido, también es necesario destacar la importancia del profesional de enfermería en el escenario internacional y nacional, cuyo ámbito de actuación incluye las relaciones interpersonales y la educación para la salud más allá del control de la enfermedad, promoviendo estilos de vida saludables, socializando conocimientos y formando sujetos críticos y autónomos en el autocuidado^(27)^. Las enfermeras son responsables de la atención prenatal en la mayoría de los países, incluidas las enfermeras obstétricas, por lo que ampliar sus competencias para la promoción de la salud y los cambios en el comportamiento sanitario es fundamental^(27)^.

Por lo tanto, la identificación de las desigualdades en el acceso a la atención sanitaria, como se ha estudiado en esta investigación, ayuda a fundamentar las intervenciones y las políticas públicas específicas destinadas a mejorar constantemente la atención ofrecida y, en consecuencia, a reducir la morbilidad y la mortalidad materna y neonatal^(28)^. A pesar de los avances en la atención obstétrica en el país y de las evidencias de este estudio sobre la alta prevalencia de orientación durante el control prenatal, cambios políticos como la reciente interrupción de la Red Cigüeña por la Red de Atención Materno-Infantil (Rami), con enfoque uniprofesional, médico-céntrico y desarticulado con otras esferas de gobierno^(29)^, en un contexto político conservador que interrumpió varias políticas para garantizar los derechos de las mujeres, junto con la desfinanciación del SUS^(12,30)^ y la pandemia, pueden haber contribuido a profundizar esas desigualdades. Esta realidad también puede ser experimentada por otros países. Por lo tanto, futuros estudios serán necesarios para acompañar continuamente este tipo de atención en el país, especialmente con la institución de la nueva estrategia gubernamental, con vistas a enfrentar estos persistentes y agravados retrocesos.

En cuanto a las limitaciones del estudio, reconocemos la posibilidad de sesgo de memoria en los datos recolectados, ya que en las entrevistas de la ENS se preguntó sobre el parto en los últimos dos años. Otra limitación es el hecho de que la muestra no fue representativa de la población amarilla e indígena, cuyos datos deben ser interpretados con cautela. Además, no fue posible evaluar avances o retrocesos en cuanto a la recepción de la mayoría de las orientaciones contempladas en la ENS 2013, con excepción de la orientación sobre referencia materna, ya que las demás orientaciones no fueron incluidas en el cuestionario de la ENS 2019. Además de no permitir el seguimiento de estos indicadores, la no inclusión de las directrices en el cuestionario refuerza una atención centrada exclusivamente en técnicas y aspectos biológicos. También apunta a la desvalorización de la educación para la salud como tecnología ligera de atención, retratada en este estudio como eje orientador de la práctica profesional para la promoción y recuperación de la salud, y la emancipación de la gestante en el proceso de atención.

## CONCLUSIÓN

Los resultados de este estudio muestran que la orientación prenatal está siendo proporcionada por los profesionales, especialmente la orientación sobre el centro de referencia de maternidad, que mostró un aumento significativo en la prevalencia de 2013 a 2019. Sin embargo, señalan desigualdades e inequidades en esta atención, como menor orientación recibida por mujeres con bajo nivel de escolaridad, pardas y negras, que viven en el Noreste, que tuvieron su atención prenatal en el sistema público de salud y, sobre todo, con menor prevalencia de orientación sobre las especificidades del ciclo embarazo-puerperio. Por lo tanto, se cree que estudios como éste, que evalúan la orientación que se da a las gestantes durante el control prenatal a nivel nacional y que abarcan un mayor número de orientaciones, pueden contribuir a una mejor evaluación de la realidad de esta atención. Además, es importante fortalecer la participación de las enfermeras en la atención integral de la salud de la mujer, con un enfoque en la promoción de la salud y un mayor compromiso en el abordaje de las desigualdades en la prestación de la orientación durante el control prenatal. Este estudio podría convertirse en un referente para futuras evaluaciones, dados los retrocesos político-programáticos en el campo de la salud de las mujeres en el país, que también puede ser la realidad en otros países, dado el creciente conservadurismo, pandemias, guerras y situaciones que ponen en mayor riesgo la salud de las mujeres y la garantía de sus derechos.
